# Supplementary material for: XRCC1 prevents toxic PARP1 trapping during DNA base excision repair
Source: Mol Cell. 2021 Jul 15;81(14):3018–3030.e5. doi: 10.1016/j.molcel.2021.05.009 (PMC8294329; doi:10.1016/j.molcel.2021.05.009)
Supplement: Document S1. Figures S1–S3 and Table S1 [file mmc1.pdf]

Molecular Cell, Volume *81*

Supplemental information

XRCC1 prevents toxic PARP1 trapping during DNA base excision repair

Annie A. Demin, Kouji Hirota, Masataka Tsuda, Marek Adamowicz, Richard Hailstone, Jan Brazina, William Gittens, Ilona Kalasova, Zhengping Shao, Shan Zha, Hiroyuki Sasanuma, Hana Hanzlikova, Shunichi Takeda, and Keith W. Caldecott

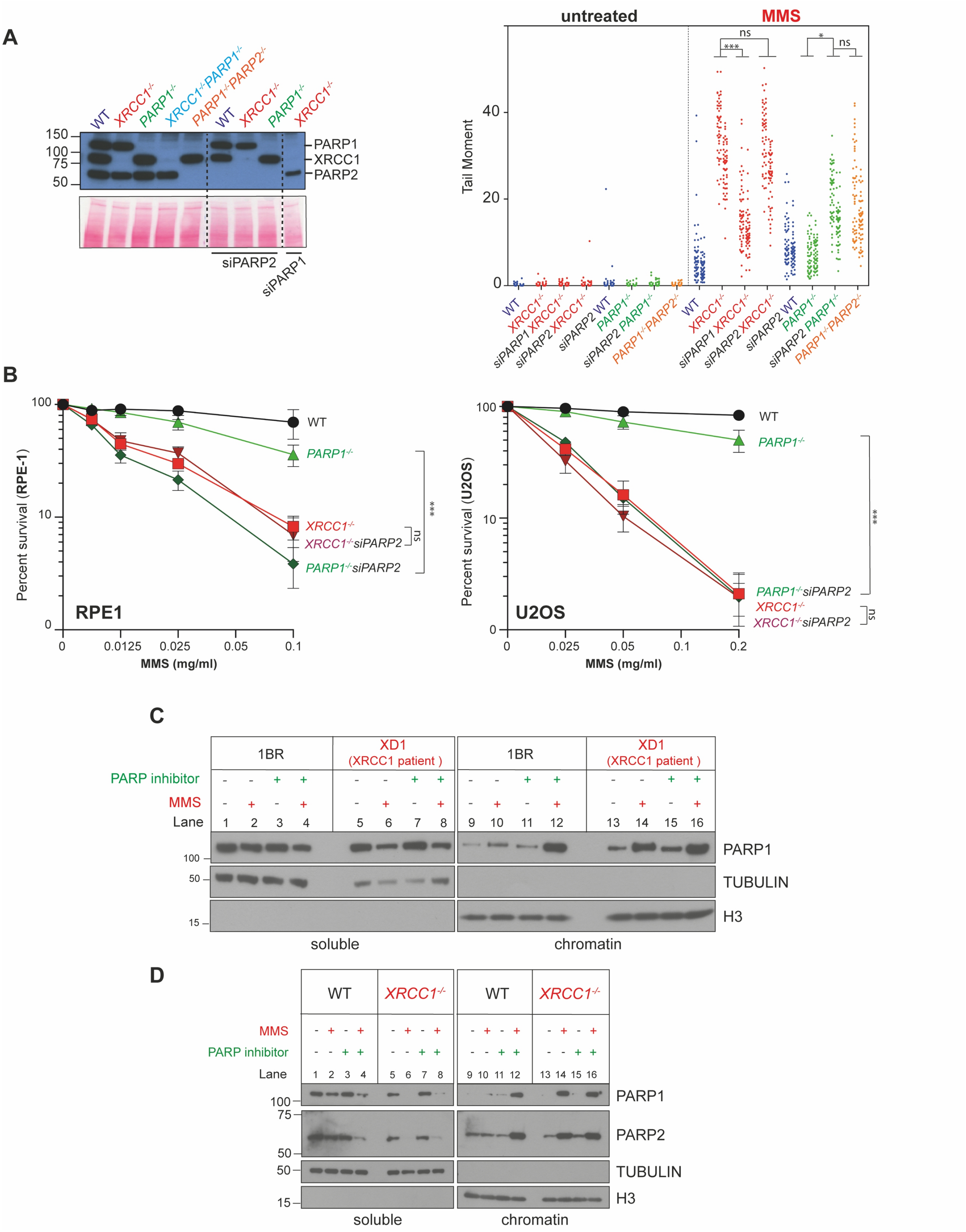

Figure S1. PARP1 but not PARP2 is responsible for the accumulation of SSBs in *XRCC1*$^{-/-}$ RPE-1 cells (see also Figure1 & Figure 2). A, *Left,* PARP1, PARP2, and XRCC1 levels in the indicated gene edited and/or siRNA treated cell lines. *Right*, Strand breaks quantified by alkaline comet assays in untreated or MMS-treated (0.1 mg/ml MMS, 15 min) wild-type (WT) and the indicated RPE1-cell lines, with or without prior treatment with PARP1/PARP2 siRNA. Data plotted are the individual comet tail moments of 50 cells per sample per experiment, for two independent experiments, with tail moments for each experiment plotted vertically and each experiment plotted side by side. Statistical significance was ascertained by one-way ANOVA, with Sidak's multiple

comparisons test (*p≤0.05; ***p≤0.001). **B**, Clonogenic survival of wild-type (WT) and the indicated gene-edited RPE-1 (*left panel*) or U2OS (*right panel*) cells after mock-treatment or treatment with PARP2 siRNA and with the indicated concentrations of MMS for 30 min, followed by incubation in drug-free medium for 10-14 days. Data are the mean (+/-SEM) of three independent experiments. Statistical significance was assessed by two-way ANOVA with Tukey's multiple comparisons test (ns, not significant; *p≤0.05; ***p≤0.001). **C,** PARP1 levels in cell-equivalent aliquots of soluble and chromatin-containing fractions of wild-type (1BR) and *XRCC1*-mutated patient (XD1) primary human fibroblasts, measured by western blotting **D**, PARP1 and PARP2 levels in cell-equivalent aliquots of soluble and chromatin-containing fractions of wild-type (WT) and *XRCC1*$^{-/-}$ RPE-1 cells, measured by western blotting. Cells were incubated or not with 10 μM PARP inhibitor (KU0058948) and/or MMS (0.1 mg/ml) for 1h as indicated, prior to subcellular fractionation.

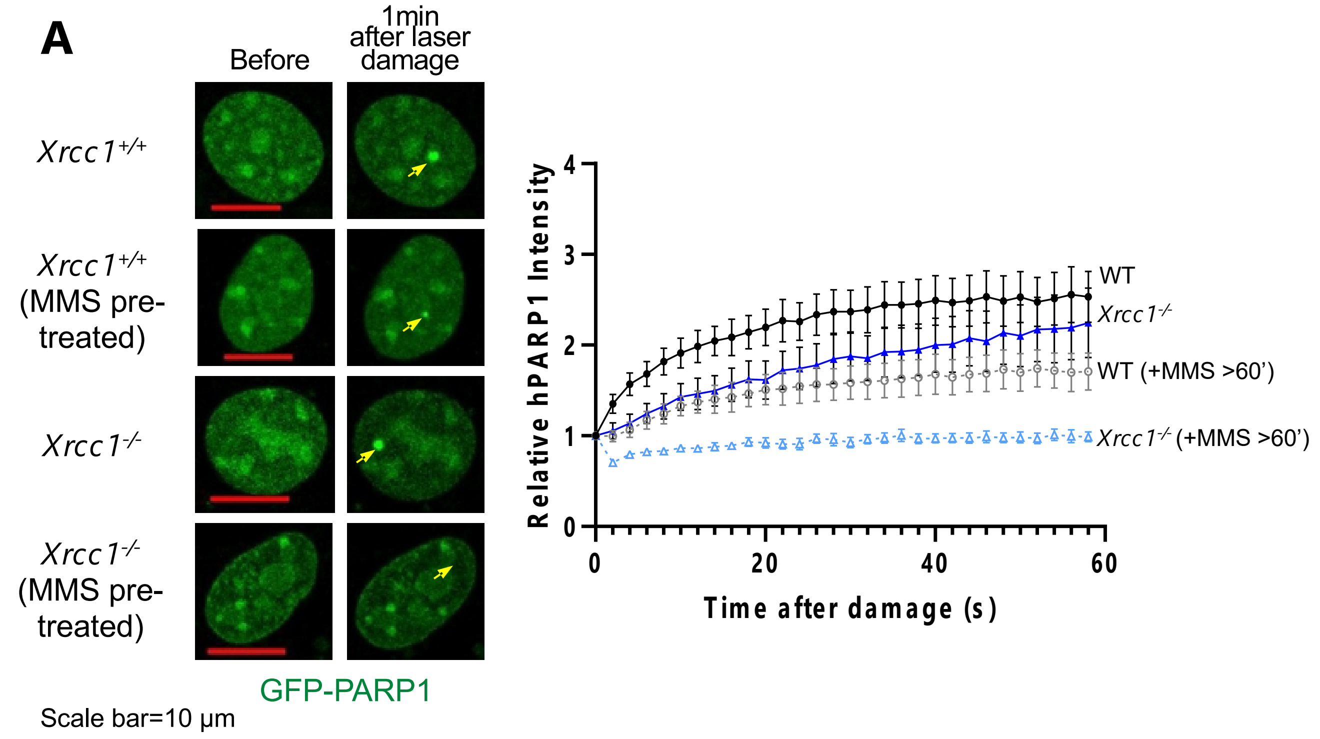

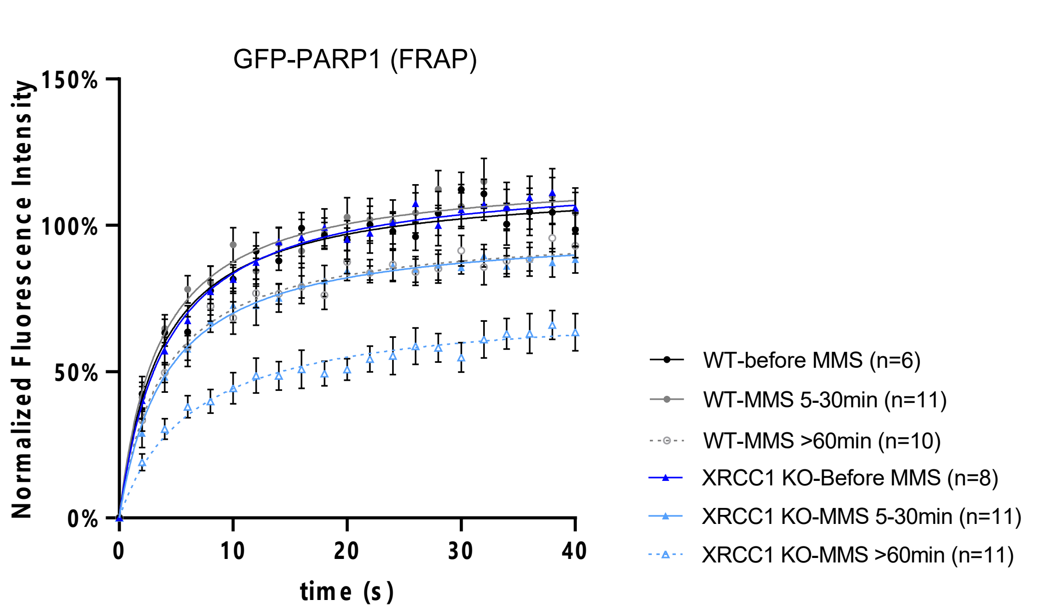

Figure S2. Reduced GFP-PARP1 mobility in MMS-treated *Xrcc1*$^{-/-}$ MEFs. A, Wild type (*Xrcc1*$^{+/+}$) and *Xrcc1*$^{-/-}$ MEFs expressing GFP-tagged PARP1 were pre-treated or not with 0.3 mg/ml MMS ≥60 min and then irradiated with 405 nm laser (yellow arrow). GFP-PARP1 recruitment at the site of damage was monitored for 60 sec following irradiations. **B,** Wild type and *Xrcc1*$^{-/-}$ MEFs expressing GFP-tagged PARP1 were pre-treated or not with 0.3 mg/ml MMS for 60 min, photobleaching by 488nm laser and monitoring of fluorescence recovery was conducted before, 5-30min after adding 0.3 mg/ml MMS, or ≥60min after adding MMS. Exchanging curves were fitted using one site ligand specific binding model using the Prism software suite. Data are representative of two independent experiments.

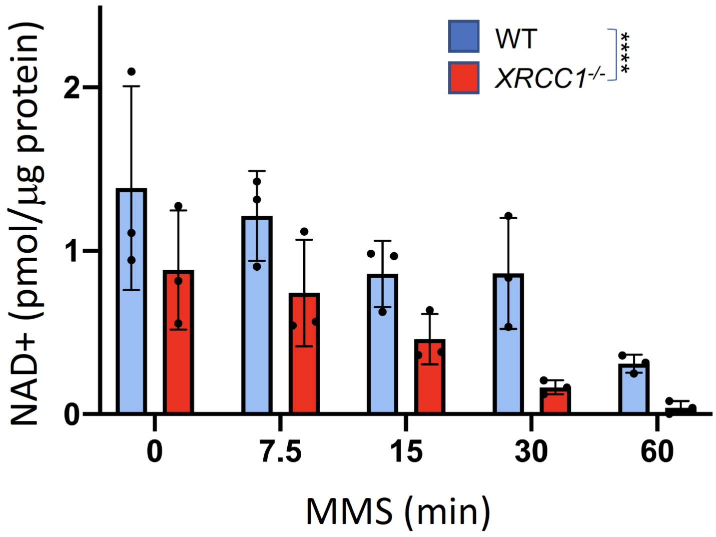

Figure S3. Increased PARP1-dependent NAD$^+$ depletion in *XRCC1$^{-/-}$* RPE1 cells, during BER (see also, Figure 4). The indicated wild type and gene-edited RPE1 cells were treated with 0.3mg/ml MMS for the indicated periods and NAD$^+$ levels present in protein extracts quantified chromogenically. Data are the mean of three independent experiments and statistical significance was assessed by 2-way ANOVA.

Supplementary Table S1; oligonucleotides employed in this study

ON-TARGETplus Human PARP1 siRNA (SMARTPool)	Dharmacon	L-006656-03-0005
ON-TARGETplus Human PARP2 siRNA (SMARTPool)	Dharmacon	L-010127-02-0020
ON-TARGETplus Non-targeting Pool	Dharmacon	D-001810-10
Double hairpin substrate 5'-GCACGGCGCATCAGCTGCAGAACAACTGCAGCTGATGCGC/deoxyU/GTGCGGATCCGGTGCAAC/iFluorT/AAGCACCGGATCC-3'	Integrated DNA Technologies (IDT)	N/A
TK6 XRCC1 knockout construct, left arm: 5'-GTAGTAAAAGACAGATGCCCACAGTCCACA-3' and 5'-CTGGCTGCTGCAGGACACGACATGGCGGAG-3'	This paper	N/A
TK6 XRCC1 knockout construct, right arm 5'-ACTCACTGTGCAGAAAATCTTCTCAAGGCA-3' and 5'-AACCACCATACCTGGCTATTATTCTTTAAA-3'	This paper	N/A
TK6 PARP1 knockout construct, left arm 5'-TGGGGAGTAGTGCTTTGTTTGGATATATCC-3' and 5'-CTGGAGAATCAAACAGACAGCAATGCTCAT-3'	This paper	N/A
TK6 PARP1 knockout construct right arm: 5'-GTAAGATCTTGGGGGCCCAGATCCCTGAAC-3' and 5'-CTTAAATTCCAAATGGCTGGCAACCTACCT-3'	This paper	N/A
β-actin (forward) 5'-GATGGTGGGCATGGGTCAGAAGGATTCC-3'	This paper	N/A
β-actin (reverse) 5'-GTCCAGGGCGAGGTAGCACAGCTTCTC-3'	This paper	N/A
