# Supplementary material for: XRCC1 prevents toxic PARP1 trapping during DNA base excision repair
Source: Mol Cell. 2021 Jul 15;81(14):3018–3030.e5. doi: 10.1016/j.molcel.2021.05.009 (PMC8294329; doi:10.1016/j.molcel.2021.05.009)
Supplement: Document S2. Article plus supplemental information [file mmc2.pdf]

Molecular Cell

Article

XRCC1 prevents toxic PARP1 trapping during DNA base excision repair

Graphical abstract

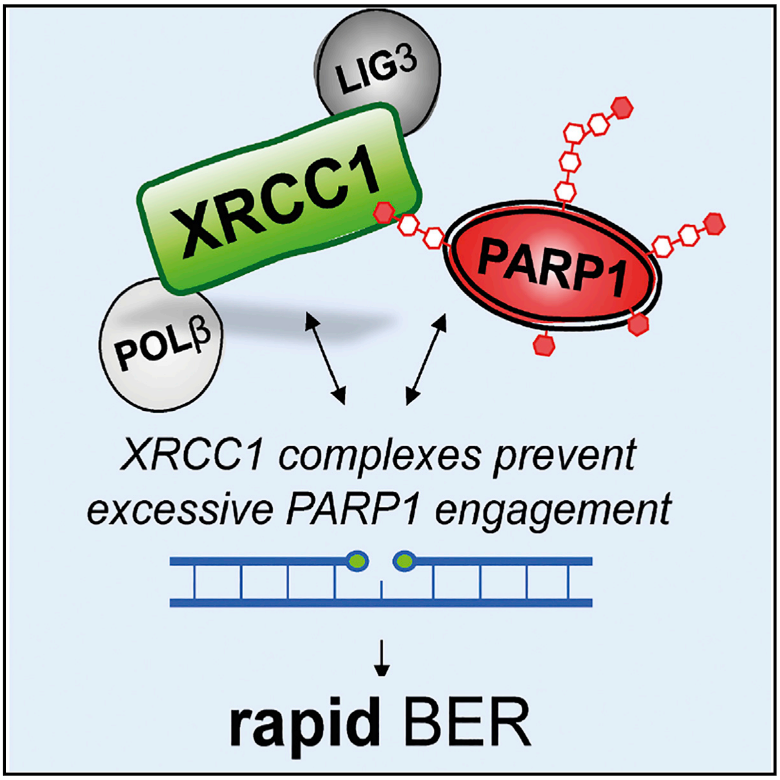

Highlights

- XRCC1 prevents endogenous PARP1 trapping during DNA base excision repair
- PARP1 trapping impedes base excision repair and increases sensitivity to base damage
- In the absence of PARP1, XRCC1 is dispensable for DNA base excision repair

Authors

Annie A. Demin, Kouji Hirota, Masataka Tsuda, ..., Hana Hanzlikova, Shunichi Takeda, Keith W. Caldecott

Correspondence

stakeda@rg.med.kyoto-u.ac.jp (S.T.),
k.w.caldecott@sussex.ac.uk (K.W.C.)

In brief

Demin et al. show that the essential role of the scaffold protein XRCC1 during DNA base excision repair is to prevent toxic "trapping" of PARP1 on SSB intermediates, which otherwise block this essential repair process and lead to increased cellular sensitivity to DNA base damage.

Demin et al., 2021, Molecular Cell *81*, 3018–3030
July 15, 2021 © 2021 The Authors. Published by Elsevier Inc.
https://doi.org/10.1016/j.molcel.2021.05.009

CellPress

CellPress OPEN ACCESS

Molecular Cell

Article

XRCC1 prevents toxic PARP1 trapping during DNA base excision repair

Annie A. Demin,[1,8] Kouji Hirota,[2,3,8] Masataka Tsuda,[2,4,8] Marek Adamowicz,[1] Richard Hailstone,[1] Jan Brazina,[1] William Gittens,[1] Ilona Kalasova,[5] Zhengping Shao,[6] Shan Zha,[6,7] Hiroyuki Sasanuma,[2] Hana Hanzlikova,[1,5] Shunichi Takeda,[2,*] and Keith W. Caldecott[1,5,9,*]

[1]Genome Damage and Stability Centre, School of Life Sciences, University of Sussex, Falmer, Brighton BN1 9RQ, UK
[2]Department of Radiation Genetics, Graduate School of Medicine, Kyoto University, Yoshidakonoe, Sakyo-ku, Kyoto 606-8501, Japan
[3]Department of Chemistry, Tokyo Metropolitan University, Minami-Osawa, Hachioji-shi, Tokyo 192-0397, Japan
[4]Program of Mathematical and Life Sciences, Graduate School of Integrated Sciences for Life, Hiroshima University, Higashi-Hiroshima 739-8526, Japan
[5]Department of Genome Dynamics, Institute of Molecular Genetics of the Czech Academy of Sciences, 142 20 Prague 4, Czech Republic
[6]Institute for Cancer Genetics, Department of Pathology and Cell Biology, College of Physicians and Surgeons, Columbia University, New York City, NY 10032, USA
[7]Division of Pediatric Oncology, Hematology and Stem Cell Transplantation, Department of Pediatrics, College of Physicians and Surgeons, Columbia University, New York, NY 10032, USA
[8]These authors contributed equally
[9]Lead contact
*Correspondence: stakeda@rg.med.kyoto-u.ac.jp (S.T.), k.w.caldecott@sussex.ac.uk (K.W.C.)
https://doi.org/10.1016/j.molcel.2021.05.009

SUMMARY

Mammalian DNA base excision repair (BER) is accelerated by poly(ADP-ribose) polymerases (PARPs) and the scaffold protein XRCC1. PARPs are sensors that detect single-strand break intermediates, but the critical role of XRCC1 during BER is unknown. Here, we show that protein complexes containing DNA polymerase β and DNA ligase III that are assembled by XRCC1 prevent excessive engagement and activity of PARP1 during BER. As a result, PARP1 becomes "trapped" on BER intermediates in XRCC1-deficient cells in a manner similar to that induced by PARP inhibitors, including in patient fibroblasts from XRCC1-mutated disease. This excessive PARP1 engagement and trapping renders BER intermediates inaccessible to enzymes such as DNA polymerase β and impedes their repair. Consequently, PARP1 deletion rescues BER and resistance to base damage in *XRCC1*$^{-/-}$ cells. These data reveal excessive PARP1 engagement during BER as a threat to genome integrity and identify XRCC1 as an "anti-trapper" that prevents toxic PARP1 activity.

INTRODUCTION

DNA base excision repair (BER) is a highly conserved pathway that is present in all organisms and required for repair of a broad range of endogenous and exogenous DNA base damage (Beard et al., 2019; Caldecott, 2020). In mammals, the canonical BER pathway involves removal of the damaged base by a DNA glycosylase, incision of the resulting abasic site by AP endonuclease (apurinic/apyrimidinic endonuclease; APE), replacement of the missing nucleotide and removal of the terminal sugar phosphate by DNA polymerase β (POLβ), and ligation of the resulting nick by DNA ligase I (LIG1) or DNA ligase III (LIG3) (Beard et al., 2019; Caldecott, 2020). The importance of BER in mammals is illustrated by the embryonic lethality observed in mice in which key components such as APE1 or POLβ are deleted (Gu et al., 1994; Xanthoudakis et al., 1996). Intriguingly, in addition to these core components, mammalian cells employ a number of additional proteins to accelerate BER, such as poly(ADP-ribose) polymerase-1 (PARP1), PARP2, and the molecular scaffold protein XRCC1 (Caldecott et al., 1994; Dantzer et al., 1999; Ding et al., 1992; Page et al., 2003; Ronson et al., 2018; Schreiber et al., 2002; Thompson et al., 1990). PARP1 and PARP2 are sensor proteins that detect and are activated by DNA strand breaks, resulting in the post-translational modification of themselves and other proteins with ADP-ribose (Amé et al., 1999; Benjamin and Gill, 1980; Hanzlikova et al., 2018; de Murcia and Ménissier de Murcia, 1994). PARP1 and PARP2 fulfil multiple roles at DNA strand breaks (Caldecott, 2014; Ray Chaudhuri and Nussenzweig, 2017). For example, PARP enzymes can modify chromatin structure directly by histone ribosylation and/or indirectly by recruitment and/or regulation of specific chromatin remodelers (Ahel et al., 2009; Chou et al., 2010; Grundy et al., 2016; de Murcia et al., 1986; Poirier et al., 1982; Polo et al., 2010). In addition, PARP activity can promote recruitment of other DNA repair proteins to accelerate repair of DNA strand breaks, of

 © 2021 The Authors. Published by Elsevier Inc.
This is an open access article under the CC BY license (http://creativecommons.org/licenses/by/4.0/).

CellPress
OPEN ACCESS

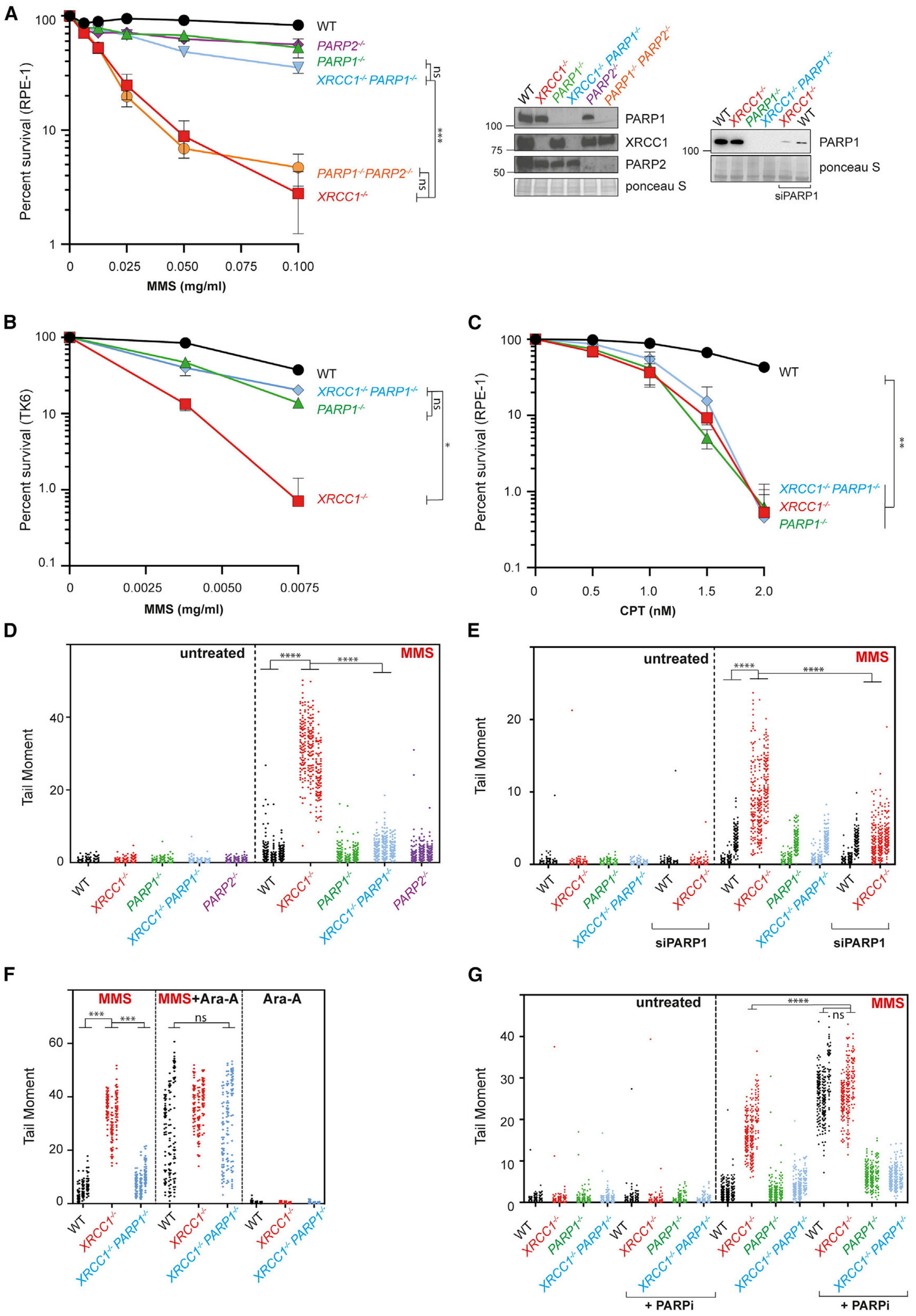

(legend on next page)

which XRCC1 and its protein partners are among the most important (El-Khamisy et al., 2003; Hanzlikova et al., 2017; Okano et al., 2003).

The importance of XRCC1 is illustrated by the observations that deletion of this gene in mouse is embryonic lethal (Tebbs et al., 1999) and that hereditary mutations in human XRCC1 result in progressive neurodegenerative disease (Hoch et al., 2017; O'Connor et al., 2018). At "direct" SSBs, such as those resulting from oxidative attack and disintegration of deoxyribose, XRCC1 binds, recruits, and stimulates DNA polynucleotide kinase phosphatase (PNKP) (Breslin et al., 2017; Hanzlikova et al., 2017; Loizou et al., 2004; Mani et al., 2007; Whitehouse et al., 2001). PNKP is ideally suited to repair of DNA strand breaks induced by oxidative damage to deoxyribose and also those induced by abortive topoisomerase I activity because it possesses the DNA kinase and DNA phosphatase activities that can restore normal 3′ and 5′ termini at such DNA breaks (Jilani et al., 1999; Karimi-Busheri et al., 1999). In contrast to direct SSBs, the role of XRCC1 during BER is less clear because PNKP is required for only a subset of the BER events that are accelerated by XRCC1 (Wiederhold et al., 2004). Moreover, although XRCC1 also interacts with and stabilizes the BER proteins POLβ (Caldecott et al., 1996; Kubota et al., 1996; Parsons et al., 2008) and LIG3 (Caldecott et al., 1994, 1995; Nash et al., 1997; Taylor et al., 1998), loss of these interactions individually only partially reduces XRCC1 functionality during BER (Breslin and Caldecott, 2009). This is in contrast to the interaction of XRCC1 with poly(ADP-ribose), which is essential for XRCC1 functionality during BER (Breslin et al., 2015). Consequently, the critical role of XRCC1 protein complexes during BER has remained elusive. Here, we have identified this role. We show that assembly of POLβ and LIG3 by XRCC1 into protein complexes is required to limit PARP1 engagement and activity during BER, which otherwise results in PARP1 "trapping" on BER intermediates in a manner reminiscent of that induced by clinical PARP inhibitors. XRCC1 is thus an endogenous "anti-trapper" that prevents toxic binding of PARP1 to SSB intermediates during BER, enabling their rapid repair and maintaining genome integrity.

RESULTS

XRCC1 suppresses PARP1-induced SSB accumulation and toxicity during BER

To address the role of XRCC1 during BER, we first examined its functional relationship with PARP1 because the ability of XRCC1 to interact directly with poly(ADP-ribose) is critical for this role (Breslin et al., 2015). To do this, we employed human RPE-1 cells in which PARP1, PARP2, and/or XRCC1 were deleted by gene editing (Hanzlikova et al., 2017; Hoch et al., 2017). As expected, RPE-1 cells lacking PARP1 alone exhibited relatively little sensitivity to the simple alkylating agent methyl methanesulfonate (MMS) compared with cells lacking XRCC1, unless PARP2 was also deleted (Figure 1A). This is consistent with the established enzymatic redundancy of PARP1 and PARP2 during BER (Ronson et al., 2018). Unexpectedly, however, deletion of PARP1 restored almost normal levels of MMS sensitivity in *XRCC1*$^{-/-}$ cells (Figure 1A). Similar results were observed in human TK6 cells in which PARP1 and/or XRCC1 were deleted by homologous recombination-mediated gene targeting, indicating that this result was not specific to RPE-1 cells or an artifact of Cas9-mediated gene editing (Figure 1B). In contrast, PARP1 deletion did not reduce the sensitivity of *XRCC1*$^{-/-}$ RPE-1 cells to camptothecin, a genotoxin that induces SSBs independently of BER by promoting the abortive activity of topoisomerase 1 (Figure 1C). These data indicate that the essential role of XRCC1 during BER is to suppress PARP1-induced cytotoxicity.

To identify the mechanism by which XRCC1 suppresses PARP1-induced cytotoxicity, we examined the effect of deleting these proteins on BER directly, using alkaline comet assays. Strikingly, PARP1 deletion prevented the appearance of elevated MMS-induced SSBs in *XRCC1*$^{-/-}$ cells, suggesting that the accumulation of these BER intermediates was the result of PARP1 (Figure 1D). Again, this was not an artifact of gene editing because depletion of PARP1 using small interfering RNA (siRNA) gave similar results (Figures 1A, right panel, and 1E). Restoration by PARP1 deletion of normal steady-state levels of MMS-induced SSBs in *XRCC1*$^{-/-}$ cells could reflect either

Figure 1. XRCC1 suppresses PARP1-dependent SSB accumulation and toxicity during BER

(A) Clonogenic survival of wild-type (WT) and gene-edited RPE-1 cells after treatment with the indicated concentrations of MMS for 30 min, followed by incubation in drug-free medium for 10–14 days. Data are the mean (±SEM) of 3 independent experiments. The level of the indicated proteins in the indicated gene-edited cell lines is shown (right). Statistical significance was assessed by two-way ANOVA with Tukey's multiple comparisons test. All cell lines were significantly different from the WT ($p \leq 0.01$), and other relevant comparisons are shown on the graph (ns, not significant; *$p \leq 0.05$, **$p \leq 0.01$, ***$p \leq 0.001$, ****$p \leq 0.0001$). See also Figures S1A and S1B.

(B), Survival of WT and the indicated gene-targeted TK6 cells after treatment with the indicated concentration of MMS for 1 h, followed by incubation in complete medium for 72 h. Cell viability was assessed by ATP assays. Data are the mean (±SEM) of 3 independent experiments, and statistics are as in (A).

(C) Clonogenic survival of WT and gene-edited RPE-1 cells following continuous treatment with the indicated concentrations of camptothecin (CPT) for 10–14 days. Data are the mean (±SEM) of 3 independent experiments, and statistics are as in (A).

(D) DNA strand breaks quantified by alkaline comet assays in the WT and the indicated gene-edited RPE-1 cell lines following treatment or no treatment with 0.1 mg/mL MMS for 15 min. Data plotted are the individual comet tail moments (an arbitrary measure of DNA strand breakage) of 100 cells per sample per experiment for 3 independent experiments, with individual cell tail moments for each experiment plotted vertically and each experiment plotted side by side. Statistical significance was ascertained by one-way ANOVA of the mean tail moments from 3 experiments with Sidak's multiple comparisons test (**$p \leq 0.01$, ***$p \leq 0.001$, ****$p \leq 0.0001$).

(E) DNA strand breaks quantified as above in the indicated RPE-1 cell lines transfected with non-targeting or PARP1 siRNA following treatment or no treatment with 0.05 mg/mL MMS for 15 min. A western blot illustrating the efficiency of PARP1 depletion is shown in (A). Data and statistics are as in (D). See also Figure S1A.

(F) DNA strand breaks quantified as above in the indicated RPE-1 cell lines following treatment or no treatment with 0.1 mg/mL MMS for 15 min in the presence or absence of 20 μM Ara-A, as indicated. A western blot shows the efficiency of PARP1 depletion (inset). Data and statistics are as in (D).

(G) DNA strand breaks quantified as above in the indicated RPE-1 cell lines following treatment or no treatment with 0.1 mg/mL MMS for 15 min in the presence of DMSO vehicle (−PARP inhibitor) or PARP inhibitor (10 μM) as indicated. Data and statistics are as in (D).

CellPress
OPEN ACCESS

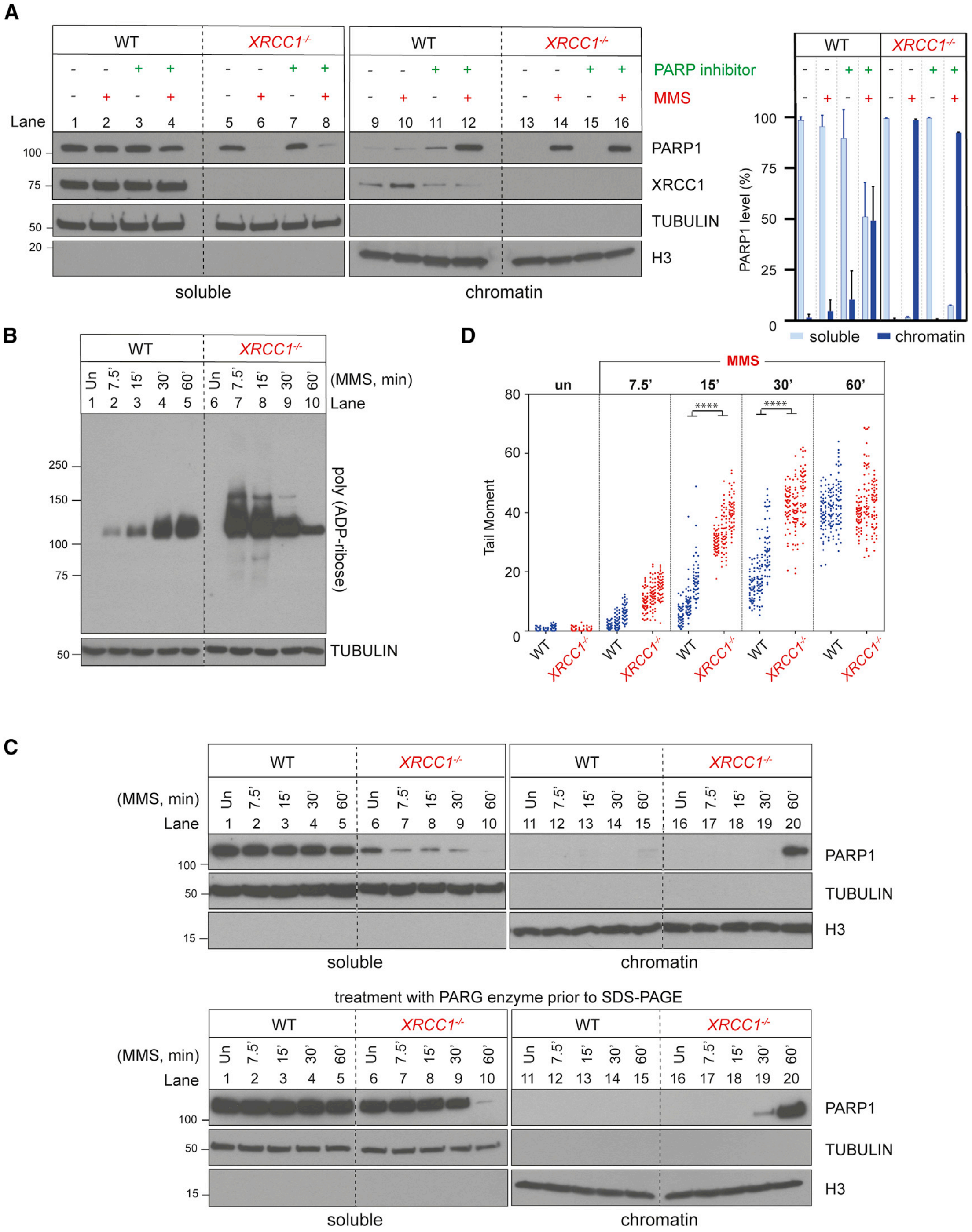

Figure 2. XRCC1 suppresses endogenous PARP1 trapping during BER

(A) PARP1 levels in cell-equivalent aliquots of soluble and chromatin-containing fractions of WT and *XRCC1*$^{-/-}$ RPE-1 cells, measured by western blotting. Cells were incubated or not with 10 μM PARP inhibitor (KU0058948) and/or MMS (0.1 mg/mL) for 1 h, as indicated, prior to subcellular fractionation. Representative immunoblots are shown on the left and quantification on the right. See also Figures S1C and S1D.

(B) Levels of PARP1 auto-ribosylation in WT and *XRCC1*$^{-/-}$ RPE-1 cells during treatment with 0.1 mg/mL MMS, detected by the poly(ADP-ribose)-specific detection reagent MABE1031.

(C) Top: PARP1 levels in cell-equivalent aliquots of soluble and chromatin-containing fractions from WT and *XRCC1*$^{-/-}$ RPE-1 cells treated for the indicated times with 0.1 mg/mL MMS. Bottom: as above, but the cell extracts were treated with recombinant PARG to remove all poly(ADP-ribose) immediately prior to SDS-PAGE.

(legend continued on next page)

reduced SSB induction during BER or increased SSB repair. To address this question, we co-incubated cells with the nucleoside analog arabinosyl adenine (Ara-A), a DNA polymerase inhibitor that can block DNA repair synthesis by promoting chain termination (Cozzarelli, 1977). As expected, co-incubation with Ara-A increased the steady-state level of MMS-induced SSBs in wild-type RPE-1 cells, consistent with inhibition of BER (Figure 1F). More importantly, Ara-A increased the level of MMS-induced SSBs in *XRCC1*$^{-/-}$/*PARP1*$^{-/-}$ cells to the same extent and to a level similar as that induced in *XRCC1*$^{-/-}$ cells (Figure 1F). These data demonstrate that PARP1 deletion prevents accumulation of MMS-induced SSBs in *XRCC1*$^{-/-}$ cells not by preventing SSB induction but by restoring normal rates of SSB repair.

In contrast to PARP1, depletion of PARP2 failed to suppress the elevated level of MMS-induced SSBs in *XRCC1*$^{-/-}$ cells or their increased sensitivity to MMS (Figures S1A and S1B). This result did not reflect inefficient PARP2 depletion because PARP2 siRNA reduced PARP2 protein to levels that were undetectable in western blots and increased MMS-induced SSBs in *PARP1*$^{-/-}$ cells as efficiently as PARP2 deletion (Figure S1A). In addition, as expected, PARP2 depletion greatly increased the sensitivity of *PARP1*$^{-/-}$ cells to MMS (Figure S1B). Collectively, these data demonstrate that the elevated accumulation of SSBs and sensitivity of *XRCC1*$^{-/-}$ cells to MMS is due to the presence of PARP1.

XRCC1 suppresses endogenous PARP1 trapping during BER

It is well established that pharmacological PARP inhibitors prolong the engagement of PARP proteins at DNA breaks and thereby slow and/or block their repair (Horton et al., 2014; Murai et al., 2012; Pommier et al., 2016). This phenomenon is called PARP trapping and underpins the clinical utility of PARP inhibitors as anti-cancer therapeutic agents (Murai et al., 2012). We therefore wanted to find out whether the PARP1-dependent accumulation of SSBs in *XRCC1*$^{-/-}$ cells reflected a similar phenomenon. Indeed, consistent with this idea, although XRCC1 deletion and PARP inhibitor increased the steady-state level of SSBs in MMS-treated RPE-1 cells, the combination of both did not increase this level above that induced by the PARP inhibitor alone (Figure 1G). We therefore examined whether MMS treatment resulted in accumulation of PARP1 in chromatin in *XRCC1*$^{-/-}$ cells because this is a measure of PARP1 trapping (Murai et al., 2012). Indeed, although PARP inhibitor was required to trigger accumulation of high levels of PARP1 in chromatin in wild-type RPE-1 cells during 1-h incubation with MMS (Figure 2A, lanes 10 and 12), most if not all cellular PARP1 accumulated in chromatin in *XRCC1*$^{-/-}$ cells during treatment with MMS, even in the absence of PARP inhibitor (Figure 2A, lanes 6 and 14). Similar results were observed when we employed primary patient fibroblasts from XRCC1-mutated disease (Hoch et al., 2017), suggesting that increased PARP1 trapping during BER is also a feature of this human genetic disease (Figure S1C). Notably, PARP2 also accumulated in the chromatin of *XRCC1*$^{-/-}$ cells during MMS treatment but, as indicated above, PARP2 did not measurably increased SSB accumulation, nor was it cytotoxic (Figure S1D). These data implicate endogenous PARP1 trapping as the source of SSB repair defects and cytotoxicity in *XRCC1*$^{-/-}$ cells during BER.

PARP1 trapping induced by pharmacological inhibitors reflects the effect of altered allosteric regulation and reduced PARP1 auto-ribosylation, which results in excessive engagement of the weakly auto-modified enzyme at SSBs and blockage of their access and repair by other enzymes (Murai et al., 2012; Satoh and Lindahl, 1992). To examine whether PARP1 might become trapped in *XRCC1*$^{-/-}$ cells by a similar mechanism, we measured the extent of PARP1 auto-ribosylation at various times during MMS treatment, using a poly(ADP-ribose)-specific detection reagent (Figure 2B). Although PARP1 auto-ribosylation was much higher in *XRCC1*$^{-/-}$ cells than in wild-type RPE-1 cells at early times (up to 15 min) after MMS addition, it declined progressively thereafter, resulting, after 60 min, in levels less than those in wild-type cells (Figure 2B). Importantly, the decline in PARP1 auto-ribosylation in *XRCC1*$^{-/-}$ RPE-1 cells was again accompanied by accumulation in chromatin of almost all cellular PARP1 (Figure 2C, top panel, lane 20). This accumulation was not simply a reflection of the high steady-state level of SSBs in *XRCC1*$^{-/-}$ cells because, by the end of the time course, high levels of SSBs were also present in wild-type RPE-1 cells (Figure 2D). Interestingly, there was a progressive reduction in PARP1 signal in the soluble fraction of *XRCC1*$^{-/-}$ cells even at early times (0–30 min) after addition of MMS despite the absence of a corresponding accumulation in chromatin at these times (Figure 2C, top panel, lanes 6–9 and 16–19). This decline reflected the effect of auto-ribosylation on detection of PARP1 by anti-PARP1 antibodies because the PARP1 signal in the soluble fraction was restored by pre-treatment of the protein samples with recombinant poly(ADP-ribose) glycohydrolase (PARG) (James et al., 2016) immediately prior to SDS-PAGE (Figure 2C, bottom panel, lanes 7–9).

The reduced PARP1 auto-ribosylation in *XRCC1*$^{-/-}$ cells 60 min after MMS treatment, compared to wild-type cells, appeared to involve reduced poly(ADP-ribose) chain length and/or branching complexity, as suggested by the faster electrophoretic mobility of the auto-ribosylated protein when fractionated extensively by SDS-PAGE and detected by a poly(ADP-ribose)-specific detection reagent (Figure 3A, compare lanes 3 and 4). That this signal reflected short chains of poly(ADP-ribose) rather than mono(ADP-ribose) was confirmed by its sensitivity to treatment with recombinant PARG immediately prior to electrophoresis (Figure 3B, compare lanes 5 and 6 with lanes 7 and 8). The reduced chain length/complexity of the poly(ADP-ribose) in *XRCC1*$^{-/-}$ cells was a result of reduced PARP1 activity rather

(D) DNA strand breaks quantified by alkaline comet assays in WT and *XRCC1*$^{-/-}$ RPE-1 cells during treatment with 0.1 mg/mL MMS. Data plotted are the individual comet tail moments (an arbitrary measure of DNA strand breakage) of 50 cells per sample per experiment, with tail moments for each experiment plotted vertically and three independent experiments plotted side by side. Statistical significance was ascertained by one-way ANOVA of the mean tail moments from 3 experiments with Sidak's multiple comparisons test (****p $\leq$ 0.0001).

CellPress
OPEN ACCESS

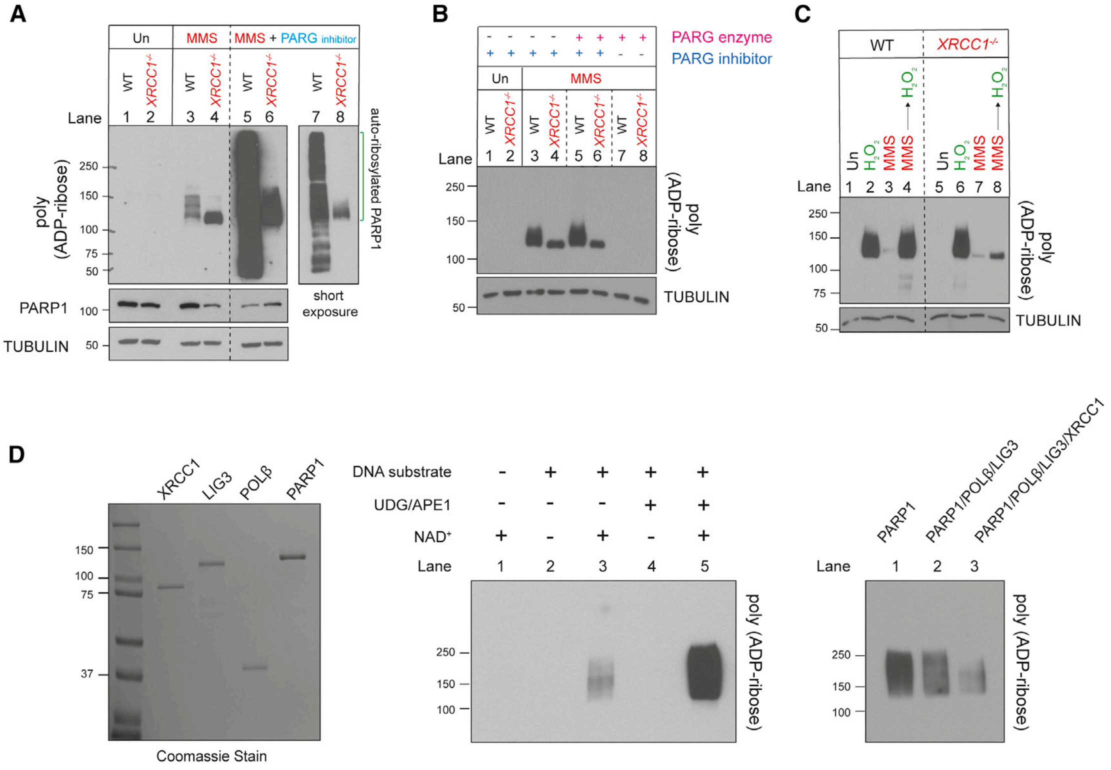

Figure 3. XRCC1 regulates PARP1 activity during BER

(A) Levels of PARP1 auto-ribosylation detected by poly(ADP-ribose)-specific detection reagent in WT and *XRCC1*$^{-/-}$ RPE-1 cells following treatment or not (untreated [Un]) with 0.1 mg/mL MMS for 1 h. Where indicated, the PARG inhibitor (PARGi) was present during the final 5 min of MMS treatment.

(B) Levels of PARP1 auto-ribosylation detected as above in total extracts prepared from Un or MMS-treated (as in A) WT and *XRCC1*$^{-/-}$ cells and following incubation of the cell extracts in the absence or presence of recombinant PARG enzyme and/or PARGi, as indicated. The PARP inhibitor was present in all cell extracts to prevent further ADP-ribosylation.

(C) Levels of PARP1 auto-ribosylation detected as above in WT and *XRCC1*$^{-/-}$ RPE-1 cells that were Un or treated with 0.1 mg/mL MMS for 1 h, with 2 mM H_2O_2 for 10 min, or sequentially with MMS and then H_2O_2.

(D) XRCC1 protein complexes regulate PARP1 activity during BER. Left: aliquots of the purified recombinant human PARP1, XRCC1-His, His-LIG3, and POLβ proteins employed here were fractionated by SDS-PAGE and stained with Coomassie brilliant blue. Center: PARP1 (0.3 μM) was incubated with or without 0.15 μM of duplex hairpin substrate harboring a site-specific uracil residue following mock treatment or pre-treatment with uracil-DNA glycosylase (UDG)/APE1 to create the SSB in the presence or absence of 10 μM NAD^+. Reaction products were fractionated by SDS-PAGE and immunoblotted with anti-poly(ADP-ribose) antibodies to detect auto-ribosylated PARP1. Note that generation of the SSB intermediate of BER (a cleaved abasic site) was required for efficient PARP1 activation. Right: PARP1 (0.3 μM) was incubated with UDG/APE1-treated substrate as above in the presence of 10 μM NAD^+ and 0.3 μM of each of the indicated recombinant proteins for 5 min at room temperature, and reaction products were processed as above.

than increased PARG activity because co-incubation with a PARG inhibitor during the final 5 min of MMS treatment failed to restore PARP1 auto-ribosylation in *XRCC1*$^{-/-}$ cells to the level observed in wild-type cells (Figure 3A, compare lanes 5–8).

Our data suggest that although PARP1 is initially engaged excessively with BER intermediates and hyperactive in *XRCC1*$^{-/-}$ cells during MMS treatment, it becomes progressively less active and unable to dissociate from BER intermediates thereafter. In support of this idea, we confirmed that the PARP1 present in *XRCC1*$^{-/-}$ cells following MMS treatment for 60 min was incapable of reactivation by a second burst of SSBs (Figure 3C). Although H_2O_2 triggered extensive PARP1 auto-ribosylation in wild-type and *XRCC1*$^{-/-}$ cells prior to MMS treatment (Figure 3C, lanes 2 and 6), it failed to do so in *XRCC1*$^{-/-}$ cells pre-treated with MMS (Figure 3C, lanes 4 and 8). This conclusion was also supported by live-cell imaging experiments showing that GFP-tagged PARP1 was unable to efficiently relocate to sites of 405-nm laser damage in *Xrcc1*$^{-/-}$ mouse embryonic fibroblasts (MEFs) if these cells were first treated with MMS (Figure S2A). In addition, fluorescence recovery after photobleaching experiments confirmed that the mobility of GFP-tagged PARP1 following MMS treatment was greatly reduced in *Xrcc1*$^{-/-}$ MEFs compared with similarly treated wild-type MEFs (Figure S2B).

XRCC1 protein complexes prevent excessive PARP1 engagement and activity during BER

Next we addressed the cause of the excessive PARP1 engagement and hyperactivity in *XRCC1*$^{-/-}$ cells during BER. This was not simply a result of the elevated SSBs in *XRCC1*$^{-/-}$ cells because, as indicated above (Figure 1D), the elevated SSBs were a consequence of excessive PARP1 engagement during BER rather than a cause. We reasoned that assembly of POLβ

and LIG3 into protein complexes by XRCC1 might be important to compete with and/or limit PARP1 engagement and activation during BER. Such a scenario would be consistent with the idea that BER intermediates are handed from one enzyme in the pathway to the next in a coordinated relay that protects the intermediates from unnecessary engagement or attack by other enzymes (Mol et al., 2000; Prasad et al., 2010; Wilson and Kunkel, 2000). Consistent with this idea, biochemical experiments employing purified human proteins confirmed that POLβ and LIG3 suppressed PARP1 activation at SSBs created by APE1 during BER and that the presence of XRCC1 greatly promoted this suppression (Figure 3D).

To further address this idea, we expressed truncated Myc-His-XRCC1$^{161–406}$ protein lacking the N-terminal and C-terminal domains that bind POLβ and LIG3 in *XRCC1$^{-/-}$* U2OS cells (Marintchev et al., 2000; Nash et al., 1997; Polo et al., 2019; Taylor et al., 1998). We employed U2OS cells rather than RPE-1 cells for these experiments because of their greater transfection efficiency. Notably, Myc-His-XRCC1$^{161–406}$ was unable to suppress the initial PARP1 hyperactivity in *XRCC1$^{-/-}$* U2OS cells (Figure 4A, compare lanes 2, 7, and 12), or its subsequent inactivation (Figure 4A, compare lanes 5, 10, and 15) and accumulation in chromatin (Figure 4B, compare lanes 12, 15, and 18). Consistent with this, Myc-His-XRCC1$^{161–406}$ was also unable to fully suppress accumulation of SSBs in *XRCC1$^{-/-}$* U2OS cells during BER (Figure 4C). The inability of Myc-His-XRCC1$^{161–406}$ to regulate PARP1 engagement during BER was not due to protein instability because the truncated protein was recruited into chromatin, although, as expected, it was unable to promote recruitment of POLβ or LIG3 (Figure 4B). These data indicate that XRCC1 regulates PARP1 engagement and activity during BER in part by assembling POLβ and LIG3 into protein complexes that can limit PARP1 access to BER intermediates.

Progressive PARP1 inactivation during BER in XRCC1-defective cells is a result of β-nicotinamide adenine dinucleotide (NAD$^+$) depletion

Because excessive PARP1 engagement and activity can result in NAD$^+$ depletion, we wanted to find out whether the subsequent decline in PARP1 activity during BER in the absence of XRCC1 protein complexes reflected NAD$^+$ exhaustion. Consistent with this idea, NAD$^+$ has been reported to be depleted more rapidly by PARP activity in XRCC1 mutant Chinese hamster ovary (CHO) cells than in wild-type cells during MMS treatment (Nakamura et al., 2003), and we confirmed that this was the case in the *XRCC1$^{-/-}$* RPE-1 cells employed here (Figure S3). To test directly whether NAD$^+$ depletion was responsible for the decline in PARP1 activity in *XRCC1$^{-/-}$* RPE-1 cells, we treated the latter with MMS for 60 min to accumulate trapped PARP1 and then incubated total cell lysates from these cells and wild-type controls with or without NAD$^+$ supplement *in vitro*. As expected, lysates prepared from MMS-treated *XRCC1$^{-/-}$* cells exhibited very little auto-ribosylated PARP1 when incubated in the absence of NAD$^+$ supplement compared with wild-type lysates incubated in parallel (Figure 4D, compare lanes 2 and 4). However, supplementation with NAD$^+$ not only stimulated ADP-ribosylation in *XRCC1$^{-/-}$* cell lysates, but it increased it above that observed in wild-type cell lysates (Figure 4D, compare lanes 2 and 4 with lanes 6 and 8). This increased ADP-ribosylation reflected the activation of PARP molecules by BER intermediates because NAD$^+$ only weakly stimulated ADP-ribosylation in cell lysates prepared from cells not pre-treated with MMS (Figure 4D, lanes 5 and 7). We conclude from these experiments that the progressive decline in PARP1 auto-ribosylation in *XRCC1$^{-/-}$* cells during BER is a result of NAD$^+$ exhaustion.

Endogenous PARP1 trapping during BER impedes POLβ recruitment into chromatin

Finally, we examined how the excessive engagement and progressive inactivation of PARP1 that occurs in *XRCC1$^{-/-}$* cells might impede BER. Treatment of wild-type RPE-1 cells with PARP inhibitor reduced XRCC1 and POLβ accumulation in chromatin, consistent with the idea that PARP1 trapping impedes BER by preventing BER enzymes from accessing SSB intermediates (Figure 5A, compare lanes 12 and 14). We therefore reasoned that the excessive engagement of PARP1 observed in *XRCC1$^{-/-}$* cells may block BER by a similar mechanism. Consistent with this, POLβ was almost undetectable in the chromatin of *XRCC1$^{-/-}$* RPE-1 cells before and after treatment with MMS (Figure 5A, lanes 15 and 16). Given the extent of this defect, we considered it unlikely that it simply reflected the effect of XRCC1 interaction on POLβ stability and/or recruitment. Indeed, PARP1 deletion fully rescued the accumulation of POLβ in chromatin in *XRCC1$^{-/-}$* cells, during BER (Figure 5A, compare lanes 11 and 12 with lanes 15 and 16 and lanes 19 and 20). Notably, PARP1 deletion also increased the accumulation of POLβ and XRCC1, even in wild-type RPE-1 cells, suggesting that PARP1 and XRCC1 protein complexes compete continuously for SSB intermediates during BER (Figure 5A, compare lanes 12 and 18).

In summary, we show here that the importance of XRCC1 during BER is to assemble protein complexes that can limit the engagement and activity of PARP1 at SSB intermediates (Figure 5B). XRCC1 is thus an endogenous PARP1 anti-trapper that safeguards genome integrity during BER by preventing PARP1 from impeding this essential DNA repair process in a manner reminiscent of anti-cancer PARP inhibitors.

DISCUSSION

DNA base excision repair (BER) is a highly conserved pathway where damaged DNA bases are excised and replaced with undamaged nucleotides using a core set of enzymes comprised of DNA glycosylase, AP endonuclease , DNA polymerase, and DNA ligase activities (Beard et al., 2019; Caldecott, 2020). In addition, human cells employ several additional proteins to accelerate BER including the SSB sensors PARP1 and/or PARP2 and the molecular scaffold protein XRCC1 (Caldecott et al., 1994; Dantzer et al., 1999; Ding et al., 1992; Page et al., 2003; Ronson et al., 2018; Schreiber et al., 2002). The importance of XRCC1 during BER is illustrated by the elevated accumulation of SSBs and cellular hypersensitivity to simple DNA base damage in cells in which XRCC1 is mutated or absent (Breslin and Caldecott, 2009; Thompson et al., 1982; Zdzienicka et al., 1992). Although it is well established that XRCC1 interacts with multiple proteins involved in BER, including the core enzymes POLβ and LIG3 (Caldecott et al., 1994, 1996; Kubota

CellPress
OPEN ACCESS

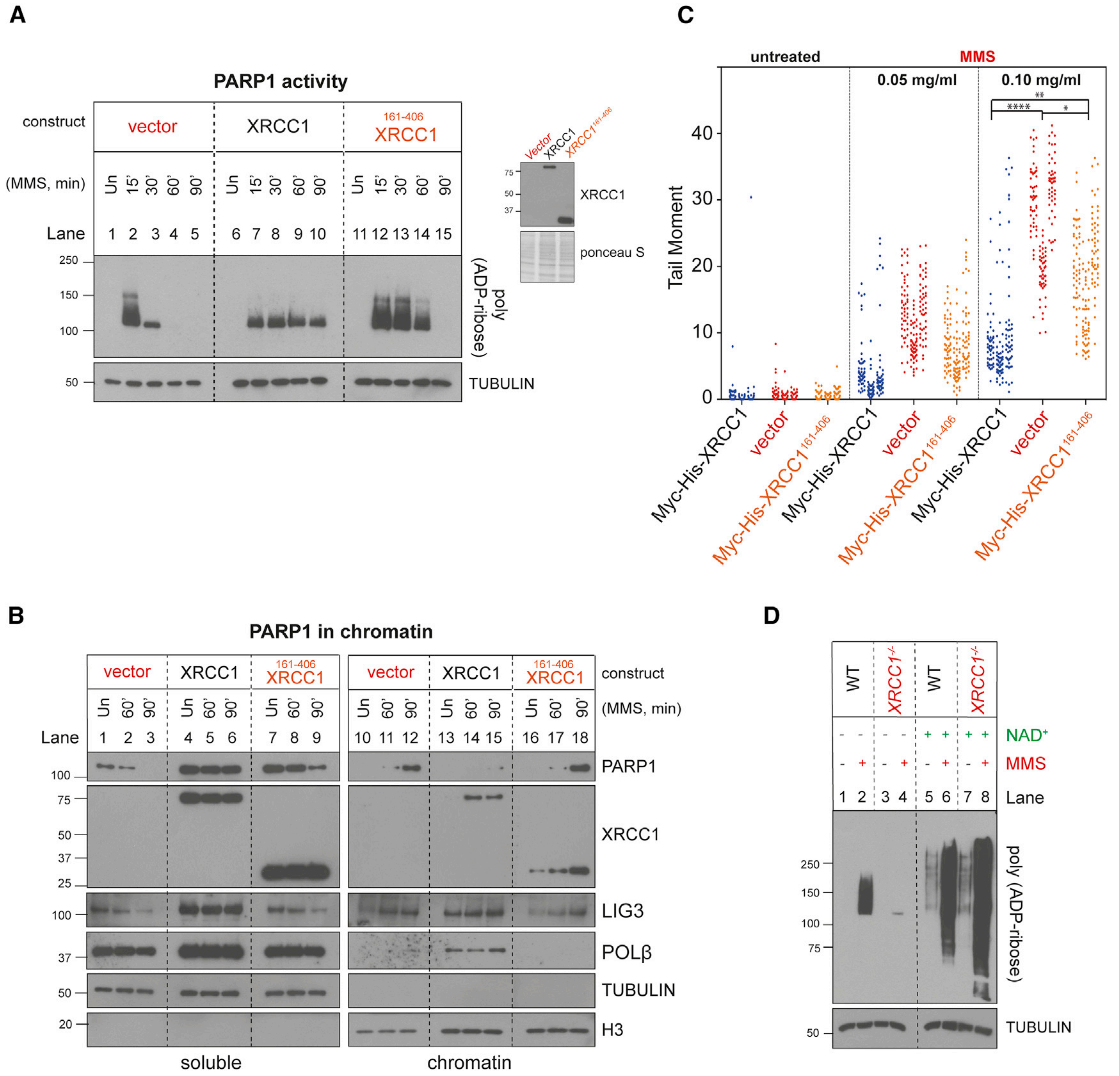

Figure 4. XRCC1 assembles protein complexes that regulate PARP1 activity, NAD$^+$ consumption, and trapping during BER

(A) Levels of PARP1 auto-ribosylation detected as above in *XRCC1*$^{-/-}$ U2OS cell lines stably transfected with empty vector or with an expression vector encoding full-length recombinant Myc-His-XRCC1 or truncated Myc-His-XRCC1$^{161-406}$ during incubation or not (Un) for the indicated times with 0.1 mg/mL MMS. The expression level of the recombinant XRCC1 proteins is shown (right).

(B) Levels of PARP1, XRCC1, LIG3, and POL β in cell-equivalent aliquots of soluble and chromatin-containing fractions from the indicated U2OS cell lines following treatment for the indicated times with 0.1 mg/mL MMS. The fractionated cell extracts were treated with recombinant PARG immediately prior to SDS-PAGE to ensure that auto-ribosylation did not obscure detection of PARP1.

(C) DNA strand breaks quantified by alkaline comet assays in the indicated U2OS cell lines following treatment with the indicated concentrations of MMS for 15 min. Data plotted are the individual comet tail moments of 50 cells per sample per experiment, with tail moments plotted vertically and each of three independent experiments plotted side by side. Statistical significance was ascertained by one-way ANOVA of the mean tail moments from 3 independent experiments with Sidak's post hoc multiple comparisons test (*$p \leq 0.05$, **$p \leq 0.01$, ****$p \leq 0.0001$).

(D) Cell extracts prepared from Un or MMS-treated (0.1 mg/mL, 60 min) WT and *XRCC1*$^{-/-}$ RPE-1 cells were incubated for 45 min in the absence or presence of 1 mM NAD$^+$, as indicated.

See also Figure S3.

et al., 1996; Nash et al., 1997), the essential role fulfilled by these interactions during BER has been unclear.

In this study, unexpectedly, our data reveal that the accumulation of SSBs and the cellular hypersensitivity to DNA base damage that are characteristic features of *XRCC1*$^{-/-}$ cells are ablated by deletion of PARP1. It is known that increased PARP1 activity resulting from elevated levels of unrepaired DNA breaks can result in cell death via NAD$^+$ depletion, defective glycolysis, and necrosis or parthanatos (Andrabi et al., 2006; Yu et al., 2006). Thus, a simple interpretation of the PARP1-induced toxicity detected here is that it is a consequence of the increased level of unrepaired SSBs in *XRCC1*$^{-/-}$ cells. However, this is not

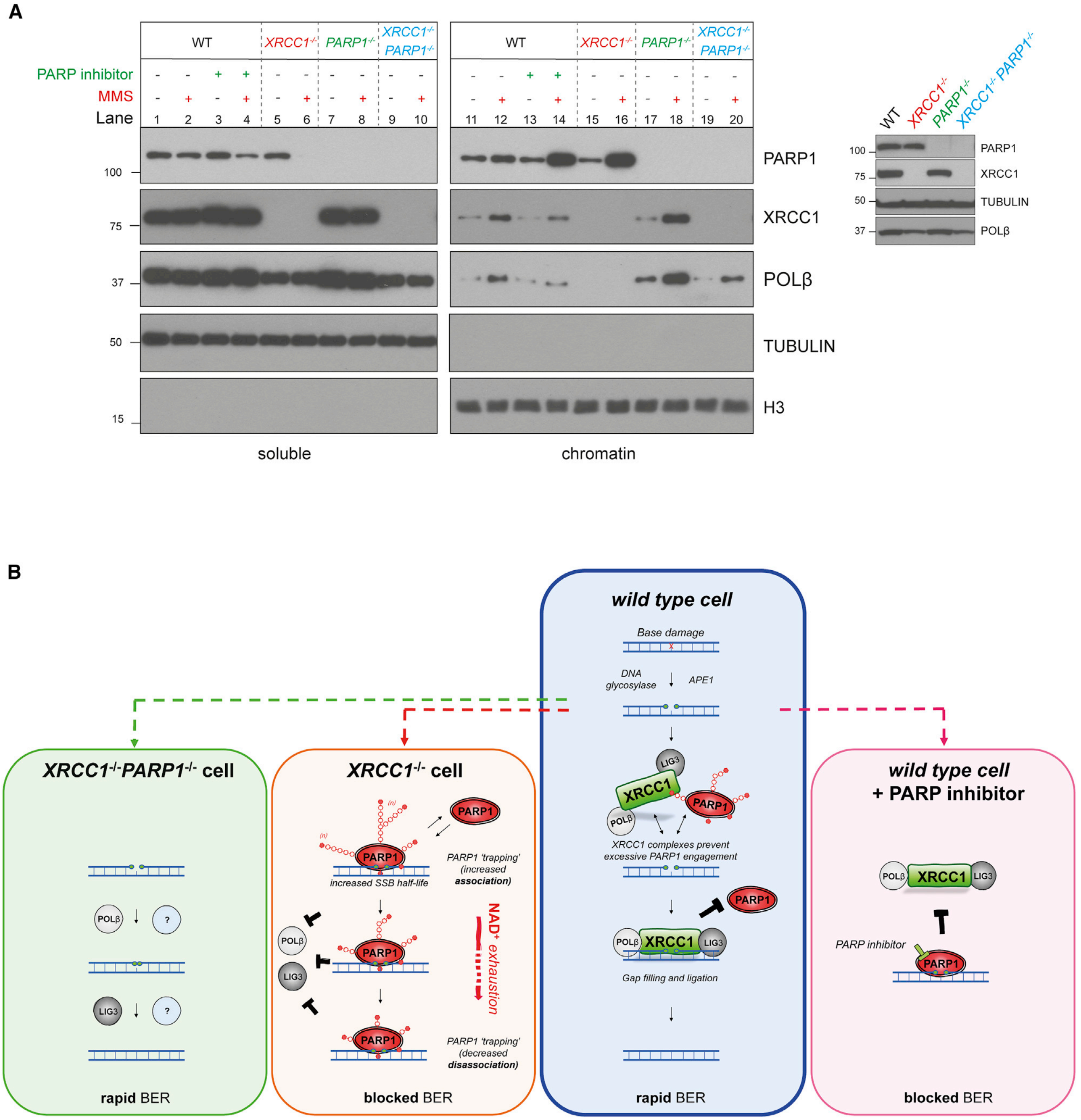

Figure 5. Endogenous PARP1 trapping impedes POLβ recruitment into chromatin during BER

(A) PARP1, XRCC1, and POLβ levels in the soluble and chromatin-containing fractions (1:4 cell equivalents, respectively) of WT and the indicated RPE-1 cell lines, measured by western blotting. Cells were pre-treated or not with the PARP inhibitor (10 μM) and/or MMS (0.1 mg/mL) for 1 h, as indicated. A western blot showing total PARP1, XRCC1, and POLβ levels in the cell lines is shown (right).

(B) A model for endogenous PARP1 trapping during BER. Blue box: in WT cells, XRCC1 protein complexes limit PARP1 engagement and activity during BER by promoting efficient hand-off of SSB intermediates to POLβ and LIG3, preventing PARP1 from impeding repair. Orange box: in *XRCC1*$^{-/-}$ cells, the absence of XRCC1 protein complexes results in excessive cycles of PARP1 association/activation at SSB intermediates, which impedes access by other BER enzymes and blocks their repair, resulting in SSB accumulation. If this scenario is sufficiently prolonged, such as at high levels of base damage, then this increased PARP1 engagement leads progressively to NAD^{+} depletion, declining PARP1 auto-ribosylation and dissociation, and accumulation of PARP1 in chromatin. PARP1 trapping in this scenario thus reflects both increased PARP1 association at SSB intermediates and subsequently decreased PARP1 dissociation, both of which impede BER in a manner reminiscent of chemical PARP inhibitors (pink box shown for comparison). Green box: additional deletion of PARP1 in *XRCC1*$^{-/-}$ cells allows access of BER intermediates by POLβ, LIG3, and/or alternative DNA repair enzymes, restoring normal rates of BER.

the case because it is increased PARP1 engagement that causes the accumulation of SSBs in *XRCC1*$^{-/-}$ cells during BER, rather than the other way around. This work reveals that the essential role of XRCC1 during BER is to prevent excessive PARP1 engagement and activity at SSB intermediates, which otherwise blocks their access and repair by other BER enzymes. This work overturns the paradigm that there is an intrinsic requirement for XRCC1 for rapid rates of BER because, in the absence of PARP1, XRCC1 is dispensable.

The excessive engagement of PARP1 during BER in the absence of XRCC1 is reminiscent of the effect of PARP inhibitors in wild-type cells. However, although PARP trapping by PARP inhibitors reflects reduced PARP1 auto-ribosylation and allosterically enhanced DNA binding resulting from chemical inhibition (Murai et al., 2012), PARP1 trapping in the absence of XRCC1 reflects the absence of BER complexes that can limit and/or regulate cycles of PARP1 association and activation. If this excessive PARP1 association and activation is prolonged sufficiently, NAD^+ becomes progressively depleted, reducing the ability of PARP1 to auto-ribosylate and dissociate from BER intermediates and promoting its accumulation in chromatin. We define both the excessive PARP1 association and subsequently reduced dissociation in this scenario as forms of PARP1 trapping, both of which directly impede BER.

The ability of XRCC1 to suppress PARP1 toxicity during BER reflects its ability to assemble BER enzymes into protein complexes because this suppression required the XRCC1 domains that interact with POLβ and LIG3. Truncated XRCC1 lacking these domains was also unable to prevent the initial hyperactivation of PARP1 during BER and its subsequent sequestration into chromatin. Truncated XRCC1 did support a small but statistically significant decrease in SSB acumulation, however, suggesting that the central DNA binding and/or poly(ADP-ribose)-binding domains that are retained in the truncated protein contribute to PARP1 regulation. This is consistent with the established requirement for these domains for proper XRCC1 function during BER (Breslin et al., 2015; Polo et al., 2019). The efficacy of XRCC1 protein complexes in regulating PARP1 most likely reflects the close proximity of POLβ and LIG3 within these complexes and that these enzymes are required consecutively during BER from the point where the SSB is created by APE1 to the final step of DNA ligation. The assembly of these enzymes into XRCC1 protein complexes may thus facilitate coordinated hand-off of SSB intermediates to POLβ and LIG3 during BER in a manner similar to that proposed for APE1 and POLβ (Mol et al., 2000; Wilson and Kunkel, 2000), reducing the opportunity for interference by PARP1. Interestingly, LIG3 possesses a "nick-sensing" zinc finger that is similar to those in PARP1 and is able to compete with and suppress PARP1 binding and activation by SSBs (Caldecott et al., 1996). It will be of interest to examine whether this zinc finger contributes to suppression of PARP1 trapping by XRCC1 complexes.

It is noteworthy that the role of XRCC1 in suppressing PARP1 trapping is not observed at all types of SSBs. For example, XRCC1 and PARP1 exhibit a simple epistatic relationship during the repair of 'direct' SSBs induced by oxidative attack of deoxyribose, in which loss of these proteins alone or together slows SSB repair to a similar extent (Hanzlikova et al., 2017; Hoch et al., 2017). This is also true for topoisomerase I-induced SSBs, as shown here with respect to the camptothecin sensitivity of RPE-1 cells in which PARP1 and/or XRCC1 are deleted. The BER intermediates that trap PARP1 are currently unknown, but likely candidates are the incised abasic sites that are created by APE1 because these are unique to this pathway and are the primary substrate of POLβ (Matsumoto and Kim, 1995). Consistent with this idea, PARP1 binds tightly to these SSBs and can even become covalently crosslinked to DNA at such sites (Prasad et al., 2014). Interestingly, PARP2 also accumulated in chromatin in *XRCC1*$^{-/-}$ cells during MMS treatment, which is expected because NAD^+ is also required for PARP2 auto-ribosylation and dissociation (Langelier et al., 2014). However, importantly, PARP2 depletion reduced neither the accumulation of SSBs in *XRCC1*$^{-/-}$ cells during BER nor their sensitivity to MMS. This may reflect a lower level of PARP2 protein/activity and/or the different mode of binding by PARP2 to BER intermediates, which lacks the high-affinity zinc-finger motifs characteristic of PARP1 (Amé et al., 1999; Langelier et al., 2014).

To explain our data, we propose the following model (Figure 5B). Although PARP1 and/or PARP2 activity is required to promote BER, most likely to regulate chromatin compaction (Ray Chaudhuri and Nussenzweig, 2017), PARP1 has a propensity to associate excessively with BER intermediates, blocking their access and repair by other BER enzymes. We define this excessive association as a form of PARP1 trapping. When NAD^+ becomes sufficiently depleted by excessive cycles of PARP1 association, the ability of PARP1 to auto-ribosylate and dissociate from BER intermediates is progressively reduced, leading to even tighter trapping and accumulation of PARP1 in chromatin. By assembling POLβ and LIG3 into protein complexes, XRCC1 can promote the molecular "hand-off" of SSB intermediates from one enzyme to the next during BER, limiting the opportunity for excessive PARP1 engagement and thereby suppressing PARP1 trapping and promoting repair. Consequently, in the absence of PARP1, XRCC1 is dispensable for rapid rates of BER because this pathway can be conducted by POLβ and LIG3 and/or other DNA repair enzymes without hindrance. Intriguingly, we also detected increased PARP1 trapping in primary human fibroblasts from XRCC1-mutated disease, and we reported recently that loss of cerebellar interneurons and cerebellar ataxia in Xrcc1-deleted mice are suppressed greatly by Parp1 deletion (Hoch et al., 2017). It will be of interest to determine whether endogenous PARP1 trapping during BER accounts for this Parp1-dependent neuropathology in Xrcc1-defective brain and disease.

Here we show that, in the absence of XRCC1, PARP1 can become excessively engaged on BER intermediates in a manner similar to that induced by anti-cancer PARP1 inhibitors, demonstrating that PARP1 trapping is a threat to normal genome integrity. We show that the essential role of XRCC1 during BER is as a PARP1 anti-trapper that regulates the engagement and activity of this enzyme, ensuring that this essential DNA repair pathway occurs rapidly and without obstruction.

Limitations of the study

We show here that PARP1 trapping is an endogenous threat to genome integrity and that it is the role of XRCC1 to prevent this from happening. Although this role requires XRCC1 to bind

poly(ADP-ribose) and assemble DNA BER enzymes into protein complexes, the precise details of how such complexes coordinate BER remain to be defined.

STAR★METHODS

Detailed methods are provided in the online version of this paper and include the following:

- KEY RESOURCES TABLE
- RESOURCE AVAILABILITY
  - Lead contact
  - Materials availability
  - Data and code availability
- EXPERIMENTAL MODEL AND SUBJECT DETAILS
  - Cell lines
- METHOD DETAILS
  - Chemicals
  - Antibodies
  - siRNA and transfection
  - Live cell imaging
  - XRCC1 protein complexes and PARP1 activity, *in vitro*.
  - Gene-targeting XRCC1 in TK6 cells
  - Alkaline comet assays
  - Cell survival assays
  - Chromatin retention assay & immunoblotting
  - NAD^+ measurements.
- QUANTIFICATION AND STATISTICAL ANALYSIS

SUPPLEMENTAL INFORMATION

Supplemental information can be found online at https://doi.org/10.1016/j.molcel.2021.05.009.

ACKNOWLEDGMENTS

This work was supported by an MRC program grant (MR/P010121/1) and ERC advanced investigator award (SIDSCA; 694996) to K.W.C., grants-in-aid from the Ministry of Education, Science, Sport and Culture (KAKENHI 25650006, 23221005, and 16H06306) to S.T.; and grants from Takeda Research and the Mitsubishi Foundation to H.S.. S.T. is also a recipient of the JSPS Core-to-Core Program, A. Advanced Research Networks. S.Z. and Z.S. were supported by NIH/NCI R01CA226852 (to S.Z.). We thank Mariano Polo, Anthony Oliver, and Laurence Pearl for providing recombinant human PARP1. This paper is dedicated to Sam Wilson.

AUTHOR CONTRIBUTIONS

K.W.C. and S.T. conceived, managed, and coordinated the study. K.W.C. wrote the manuscript, and all authors edited the manuscript. A.A.D. conceived and/or conducted all MMS and biochemical experiments except those involving TK6 cells, which were conducted by K.H., M.T., and H.S.; those involving MEFs, which were conducted by Z.S. and S.Z.; those involving NAD^+ measurements, which were conducted by I.K. under the guidance and supervision of H.H.; and CPT experiments, which were conducted by W.G. M.A. generated U2OS cells expressing recombinant XRCC1, and R.H. expressed and purified recombinant human XRCC1, POLβ, and LIG3. J.B. generated unpublished gene-edited RPE-1 cell lines.

DECLARATION OF INTERESTS

The authors declare no competing interests.

Received: December 7, 2020
Revised: March 8, 2021
Accepted: May 10, 2021
Published: June 7, 2021

REFERENCES

Ahel, D., Horejsí, Z., Wiechens, N., Polo, S.E., Garcia-Wilson, E., Ahel, I., Flynn, H., Skehel, M., West, S.C., Jackson, S.P., et al. (2009). Poly(ADP-ribose)-dependent regulation of DNA repair by the chromatin remodeling enzyme ALC1. Science *325*, 1240–1243.

Amé, J.-C., Rolli, V., Schreiber, V., Niedergang, C., Apiou, F., Decker, P., Muller, S., Höger, T., Ménissier-de Murcia, J., and de Murcia, G. (1999). PARP-2, A novel mammalian DNA damage-dependent poly(ADP-ribose) polymerase. J. Biol. Chem. *274*, 17860–17868.

Andrabi, S.A., Kim, N.S., Yu, S.-W., Wang, H., Koh, D.W., Sasaki, M., Klaus, J.A., Otsuka, T., Zhang, Z., Koehler, R.C., et al. (2006). Poly(ADP-ribose) (PAR) polymer is a death signal. Proc. Natl. Acad. Sci. USA *103*, 18308–18313.

Baker, J.L., Faustoferri, R.C., Quivey, R.G., and Jr. (2016). A Modified Chromogenic Assay for Determination of the Ratio of Free Intracellular NAD+/NADH in Streptococcus mutans. Bio. Protoc. *6*, e1902.

Beard, W.A., Horton, J.K., Prasad, R., and Wilson, S.H. (2019). Eukaryotic Base Excision Repair: New Approaches Shine Light on Mechanism. Annu. Rev. Biochem. *88*, 137–162.

Benjamin, R.C., and Gill, D.M. (1980). ADP-ribosylation in mammalian cell ghosts. Dependence of poly(ADP-ribose) synthesis on strand breakage in DNA. J. Biol. Chem. *255*, 10493–10501.

Breslin, C., and Caldecott, K.W. (2009). DNA 3′-phosphatase activity is critical for rapid global rates of single-strand break repair following oxidative stress. Mol. Cell Biol. *29*, 4653–4662.

Breslin, C., Clements, P.M., El-Khamisy, S.F., Petermann, E., Iles, N., and Caldecott, K.W. (2006). Measurement of chromosomal DNA single-strand breaks and replication fork progression rates. Methods Enzymol. *409*, 410–425.

Breslin, C., Hornyak, P., Ridley, A., Rulten, S.L., Hanzlikova, H., Oliver, A.W., and Caldecott, K.W. (2015). The XRCC1 phosphate-binding pocket binds poly (ADP-ribose) and is required for XRCC1 function. Nucleic Acids Res. *43*, 6934–6944.

Breslin, C., Mani, R.S., Fanta, M., Hoch, N., Weinfeld, M., and Caldecott, K.W. (2017). The RIR motif in the scaffold protein XRCC1 mediates a low-affinity interaction with polynucleotide kinase/phosphatase (PNKP) during DNA single-strand break repair. J. Biol. Chem. *292*, 16024–16031.

Caldecott, K.W. (2014). Protein ADP-ribosylation and the cellular response to DNA strand breaks. DNA Repair *19*, 108–113.

Caldecott, K.W. (2020). Mammalian DNA base excision repair: Dancing in the moonlight. DNA Repair (Amst.) *93*, 102921.

Caldecott, K.W., McKeown, C.K., Tucker, J.D., Ljungquist, S., and Thompson, L.H. (1994). An interaction between the mammalian DNA repair protein XRCC1 and DNA ligase III. Mol. Cell. Biol. *14*, 68–76.

Caldecott, K.W., Tucker, J.D., Stanker, L.H., and Thompson, L.H. (1995). Characterization of the XRCC1-DNA ligase III complex in vitro and its absence from mutant hamster cells. Nucleic Acids Res. *23*, 4836–4843.

Caldecott, K.W., Aoufouchi, S., Johnson, P., and Shall, S. (1996). XRCC1 polypeptide interacts with DNA polymerase beta and possibly poly (ADP-ribose) polymerase, and DNA ligase III is a novel molecular 'nick-sensor' in vitro. Nucleic Acids Res. *24*, 4387–4394.

Chou, D.M., Adamson, B., Dephoure, N.E., Tan, X., Nottke, A.C., Hurov, K.E., Gygi, S.P., Colaiácovo, M.P., and Elledge, S.J. (2010). A chromatin localization screen reveals poly (ADP ribose)-regulated recruitment of the repressive polycomb and NuRD complexes to sites of DNA damage. Proc. Natl. Acad. Sci. USA *107*, 18475–18480.

Cozzarelli, N.R. (1977). The mechanism of action of inhibitors of DNA synthesis. Annu. Rev. Biochem. *46*, 641–668.

CellPress
OPEN ACCESS

Dantzer, F., Schreiber, V., Niedergang, C., Trucco, C., Flatter, E., De La Rubia, G., Oliver, J., Rolli, V., Ménissier-de Murcia, J., and de Murcia, G. (1999). Involvement of poly(ADP-ribose) polymerase in base excision repair. Biochimie *81*, 69–75.

de Murcia, G., and Ménissier de Murcia, J. (1994). Poly(ADP-ribose) polymerase: a molecular nick-sensor. Trends Biochem. Sci. *19*, 172–176.

de Murcia, G., Huletsky, A., Lamarre, D., Gaudreau, A., Pouyet, J., Daune, M., and Poirier, G.G. (1986). Modulation of chromatin superstructure induced by poly(ADP-ribose) synthesis and degradation. J. Biol. Chem. *261*, 7011–7017.

Ding, R., Pommier, Y., Kang, V.H., and Smulson, M. (1992). Depletion of poly(ADP-ribose) polymerase by antisense RNA expression results in a delay in DNA strand break rejoining. J. Biol. Chem. *267*, 12804–12812.

El-Khamisy, S.F., Masutani, M., Suzuki, H., and Caldecott, K.W. (2003). A requirement for PARP-1 for the assembly or stability of XRCC1 nuclear foci at sites of oxidative DNA damage. Nucleic Acids Res. *31*, 5526–5533.

Grundy, G.J., Polo, L.M., Zeng, Z., Rulten, S.L., Hoch, N.C., Paomephan, P., Xu, Y., Sweet, S.M., Thorne, A.W., Oliver, A.W., et al. (2016). PARP3 is a sensor of nicked nucleosomes and monoribosylates histone H2B(Glu2). Nat. Commun. *7*, 12404.

Gu, H., Marth, J.D., Orban, P.C., Mossmann, H., and Rajewsky, K. (1994). Deletion of a DNA polymerase beta gene segment in T cells using cell type-specific gene targeting. Science *265*, 103–106.

Hanzlikova, H., Gittens, W., Krejcikova, K., Zeng, Z., and Caldecott, K.W. (2017). Overlapping roles for PARP1 and PARP2 in the recruitment of endogenous XRCC1 and PNKP into oxidized chromatin. Nucleic Acids Res. *45*, 2546–2557.

Hanzlikova, H., Kalasova, I., Demin, A.A., Pennicott, L.E., Cihlarova, Z., and Caldecott, K.W. (2018). The Importance of Poly(ADP-Ribose) Polymerase as a Sensor of Unligated Okazaki Fragments during DNA Replication. Mol. Cell *71*, 319–331.e3.

Hoch, N.C., Hanzlikova, H., Rulten, S.L., Tétreault, M., Komulainen, E., Ju, L., Hornyak, P., Zeng, Z., Gittens, W., Rey, S.A., et al.; Care4Rare Canada Consortium (2017). XRCC1 mutation is associated with PARP1 hyperactivation and cerebellar ataxia. Nature *541*, 87–91.

Horton, J.K., Stefanick, D.F., Prasad, R., Gassman, N.R., Kedar, P.S., and Wilson, S.H. (2014). Base excision repair defects invoke hypersensitivity to PARP inhibition. Mol. Cancer Res. *12*, 1128–1139.

James, D.I., Smith, K.M., Jordan, A.M., Fairweather, E.E., Griffiths, L.A., Hamilton, N.S., Hitchin, J.R., Hutton, C.P., Jones, S., Kelly, P., et al. (2016). First-in-class chemical probes against poly(ADP-ribose) glycohydrolase (PARG) inhibit DNA repair with differential pharmacology to olaparib. ACS Chem. Biol. *11*, 3179–3190.

Jilani, A., Ramotar, D., Slack, C., Ong, C., Yang, X.M., Scherer, S.W., and Lasko, D.D. (1999). Molecular cloning of the human gene, PNKP, encoding a polynucleotide kinase 3′-phosphatase and evidence for its role in repair of DNA strand breaks caused by oxidative damage. J. Biol. Chem. *274*, 24176–24186.

Karimi-Busheri, F., Daly, G., Robins, P., Canas, B., Pappin, D.J.C., Sgouros, J., Miller, G.G., Fakhrai, H., Davis, E.M., Le Beau, M.M., and Weinfeld, M. (1999). Molecular characterization of a human DNA kinase. J. Biol. Chem. *274*, 24187–24194.

Kubota, Y., Nash, R.A., Klungland, A., Schär, P., Barnes, D.E., and Lindahl, T. (1996). Reconstitution of DNA base excision-repair with purified human proteins: interaction between DNA polymerase beta and the XRCC1 protein. EMBO J. *15*, 6662–6670.

Langelier, M.-F., Riccio, A.A., and Pascal, J.M. (2014). PARP-2 and PARP-3 are selectively activated by 5′ phosphorylated DNA breaks through an allosteric regulatory mechanism shared with PARP-1. Nucleic Acids Res. *42*, 7762–7775.

Loizou, J.I., El-Khamisy, S.F., Zlatanou, A., Moore, D.J., Chan, D.W., Qin, J., Sarno, S., Meggio, F., Pinna, L.A., and Caldecott, K.W. (2004). The protein kinase CK2 facilitates repair of chromosomal DNA single-strand breaks. Cell *117*, 17–28.

Mani, R.S., Fanta, M., Karimi-Busheri, F., Silver, E., Virgen, C.A., Caldecott, K.W., Cass, C.E., and Weinfeld, M. (2007). XRCC1 stimulates polynucleotide kinase by enhancing its damage discrimination and displacement from DNA repair intermediates. J. Biol. Chem. *282*, 28004–28013.

Marintchev, A., Robertson, A., Dimitriadis, E.K., Prasad, R., Wilson, S.H., and Mullen, G.P. (2000). Domain specific interaction in the XRCC1-DNA polymerase β complex. Nucleic Acids Res. *28*, 2049–2059.

Matsumoto, Y., and Kim, K. (1995). Excision of deoxyribose phosphate residues by DNA polymerase beta during DNA repair. Science *269*, 699–702.

Mol, C.D., Izumi, T., Mitra, S., and Tainer, J.A. (2000). DNA-bound structures and mutants reveal abasic DNA binding by APE1 and DNA repair coordination. Nature *403*, 451–456.

Murai, J., Huang, S.Y., Das, B.B., Renaud, A., Zhang, Y., Doroshow, J.H., Ji, J., Takeda, S., and Pommier, Y. (2012). Trapping of PARP1 and PARP2 by Clinical PARP Inhibitors. Cancer Res. *72*, 5588–5599.

Nakamura, J., Asakura, S., Hester, S.D., de Murcia, G., Caldecott, K.W., and Swenberg, J.A. (2003). Quantitation of intracellular NAD(P)H can monitor an imbalance of DNA single strand break repair in base excision repair deficient cells in real time. Nucleic Acids Res. *31*, e104.

Nash, R.A., Caldecott, K.W., Barnes, D.E., and Lindahl, T. (1997). XRCC1 protein interacts with one of two distinct forms of DNA ligase III. Biochemistry *36*, 5207–5211.

O'Connor, E., Vandrovcova, J., Bugiardini, E., Chelban, V., Manole, A., Davagnanam, I., Wiethoff, S., Pittman, A., Lynch, D.S., Efthymiou, S., et al. (2018). Mutations in XRCC1 cause cerebellar ataxia and peripheral neuropathy. J. Neurol. Neurosurg. Psychiatry *89*, 1230–1232.

Okano, S., Lan, L., Caldecott, K.W., Mori, T., and Yasui, A. (2003). Spatial and temporal cellular responses to single-strand breaks in human cells. Mol. Cell. Biol. *23*, 3974–3981.

Page, F.L., Schreiber, V., Dherin, C., de Murcia, G., and Boiteux, S. (2003). Poly(ADP-ribose) polymerase-1 (PARP-1) is required in murine cell lines for base excision repair of oxidative DNA damage in the absence of DNA polymerase beta. J. Biol. Chem. *278*, 18471–18477.

Parsons, J.L., Tait, P.S., Finch, D., Dianova, I.I., Allinson, S.L., and Dianov, G.L. (2008). CHIP-mediated degradation and DNA damage-dependent stabilization regulate base excision repair proteins. Mol. Cell *29*, 477–487.

Poirier, G.G., de Murcia, G., Jongstra-Bilen, J., Niedergang, C., and Mandel, P. (1982). Poly(ADP-ribosyl)ation of polynucleosomes causes relaxation of chromatin structure. Proc. Natl. Acad. Sci. USA *79*, 3423–3427.

Polo, S.E., Kaidi, A., Baskcomb, L., Galanty, Y., and Jackson, S.P. (2010). Regulation of DNA-damage responses and cell-cycle progression by the chromatin remodelling factor CHD4. EMBO J. *29*, 3130–3139.

Polo, L.M., Xu, Y., Hornyak, P., Garces, F., Zeng, Z., Hailstone, R., Matthews, S.J., Caldecott, K.W., Oliver, A.W., and Pearl, L.H. (2019). Efficient Single-Strand Break Repair Requires Binding to Both Poly(ADP-Ribose) and DNA by the Central BRCT Domain of XRCC1. Cell Rep. *26*, 573–581.e5.

Pommier, Y., O'Connor, M.J., and de Bono, J. (2016). Laying a trap to kill cancer cells: PARP inhibitors and their mechanisms of action. Sci. Transl. Med. *8*, 362ps17.

Prasad, R., Shock, D.D., Beard, W.A., and Wilson, S.H. (2010). Substrate channeling in mammalian base excision repair pathways: passing the baton. J. Biol. Chem. *285*, 40479–40488.

Prasad, R., Horton, J.K., Chastain, P.D., Gassman, N.R., Freudenthal, B.D., Hou, E.W., and Wilson, S.H. (2014). Suicidal cross-linking of PARP-1 to AP site intermediates in cells undergoing base excision repair. Nucleic Acids Res. *42*, 6337–6351.

Ray Chaudhuri, A., and Nussenzweig, A. (2017). The multifaceted roles of PARP1 in DNA repair and chromatin remodelling. Nat. Rev. Mol. Cell Biol. *18*, 610–621.

Ronson, G.E., Piberger, A.L., Higgs, M.R., Olsen, A.L., Stewart, G.S., McHugh, P.J., Petermann, E., and Lakin, N.D. (2018). PARP1 and PARP2 stabilise replication forks at base excision repair intermediates through Fbh1-dependent Rad51 regulation. Nat. Commun. *9*, 746.

Satoh, M.S., and Lindahl, T. (1992). Role of poly(ADP-ribose) formation in DNA repair. Nature *356*, 356–358.

Schreiber, V., Amé, J.-C., Dollé, P., Schultz, I., Rinaldi, B., Fraulob, V., Ménissier-de Murcia, J., and de Murcia, G. (2002). Poly(ADP-ribose) polymerase-2 (PARP-2) is required for efficient base excision DNA repair in association with PARP-1 and XRCC1. J. Biol. Chem. *277*, 23028–23036.

Shao, Z., Lee, B.J., Rouleau-Turcotte, É., Langelier, M.-F., Lin, X., Estes, V.M., Pascal, J.M., and Zha, S. (2020). Clinical PARP inhibitors do not abrogate PARP1 exchange at DNA damage sites in vivo. Nucleic Acids Res. *48*, 9694–9709.

Taylor, R.M., Wickstead, B., Cronin, S., and Caldecott, K.W. (1998). Role of a BRCT domain in the interaction of DNA ligase III-α with the DNA repair protein XRCC1. Curr. Biol. *8*, 877–880.

Tebbs, R.S., Flannery, M.L., Meneses, J.J., Hartmann, A., Tucker, J.D., Thompson, L.H., Cleaver, J.E., and Pedersen, R.A. (1999). Requirement for the Xrcc1 DNA base excision repair gene during early mouse development. Dev. Biol. *208*, 513–529.

Thompson, L.H., Brookman, K.W., Dillehay, L.E., Carrano, A.V., Mazrimas, J.A., Mooney, C.L., and Minkler, J.L. (1982). A CHO-cell strain having hypersensitivity to mutagens, a defect in DNA strand-break repair, and an extraordinary baseline frequency of sister-chromatid exchange. Mutation Research *95*, 427–440.

Thompson, L.H., Brookman, K.W., Jones, N.J., Allen, S.A., and Carrano, A.V. (1990). Molecular cloning of the human XRCC1 gene, which corrects defective DNA strand break repair and sister chromatid exchange. Mol. Cell. Biol. *10*, 6160–6171.

Tsuda, M., Cho, K., Ooka, M., Shimizu, N., Watanabe, R., Yasui, A., Nakazawa, Y., Ogi, T., Harada, H., Agama, K., et al. (2017). ALC1/CHD1L, a chromatin-remodeling enzyme, is required for efficient base excision repair. PLoS ONE *12*, e0188320.

Whitehouse, C.J., Taylor, R.M., Thistlethwaite, A., Zhang, H., Karimi-Busheri, F., Lasko, D.D., Weinfeld, M., and Caldecott, K.W. (2001). XRCC1 stimulates human polynucleotide kinase activity at damaged DNA termini and accelerates DNA single-strand break repair. Cell *104*, 107–117.

Wiederhold, L., Leppard, J.B., Kedar, P., Karimi-Busheri, F., Rasouli-Nia, A., Weinfeld, M., Tomkinson, A.E., Izumi, T., Prasad, R., Wilson, S.H., et al. (2004). AP endonuclease-independent DNA base excision repair in human cells. Mol. Cell *15*, 209–220.

Wilson, S.H., and Kunkel, T.A. (2000). Passing the baton in base excision repair. Nat. Struct. Biol. *7*, 176–178.

Xanthoudakis, S., Smeyne, R.J., Wallace, J.D., and Curran, T. (1996). The redox/DNA repair protein, Ref-1, is essential for early embryonic development in mice. Proc. Natl. Acad. Sci. USA *93*, 8919–8923.

Yu, S.-W., Andrabi, S.A., Wang, H., Kim, N.S., Poirier, G.G., Dawson, T.M., and Dawson, V.L. (2006). Apoptosis-inducing factor mediates poly(ADP-ribose) (PAR) polymer-induced cell death. Proc. Natl. Acad. Sci. USA *103*, 18314–18319.

Zdzienicka, M.Z., Schans, G.P., van der, Natarajan, A.T., Thompson, L.H., Neuteboom, I., and Simons, J.W. (1992). A Chinese hamster ovary cell mutant (EM-C11) with sensitivity to simple alkylating agents and a very high level of sister chromatid exchanges. Mutagenesis *7*, 265–269.

STAR★METHODS

KEY RESOURCES TABLE

REAGENT or RESOURCE	SOURCE	IDENTIFIER
Antibodies		
Mouse monoclonal anti-PARP1	Santa Cruz	Cat#sc-8007; RRID:AB_628105
Rabbit polyclonal anti-XRCC1	Novus	Cat#NBP1-87154; RRID:AB_11029388
Rabbit anti-poly-ADP-ribose binding reagent	Millipore	Cat#MABE1031; RRID:AB_2665467
Mouse monoclonal anti-alpha-Tubulin	Sigma-Aldrich	Cat#T6074; RRID:AB_477582
Rabbit polyclonal anti-histone H3	Abcam	Cat#ab1791; RRID:AB_302613
Rabbit polyclonal anti-DNA Polymerase Beta	Millipore	Cat#6C0087; RRID:N/A
Rabbit sera anti-DNA ligase III	Tomas Lindahl	TL25; RRID:N/A
Rabbit polyclonal anti-PARP2	Active Motif	Cat#39743; RRID:AB_2793328
Bacterial and virus strains		
BL21 (DE3)	NEB	Cat#C2527H
Chemicals, peptides, and recombinant proteins		
Poly (ADP ribose) polymerase (PARP) inhibitor, KU0058948 hydrochloride	Axon	Cat#2001; CAS: 763111-49-5
Poly (ADP ribose) glycohydrolase (PARG) inhibitor	Tocris	Cat#5952; CAS: 1945950-21-9
Methyl methanesulfonate (MMS)	Sigma-Aldrich	Cat#129925
cOmplete, EDTA-free Protease Inhibitor Cocktail	Roche	Cat#11873580001
Adenine 9-β-D-arabinofuranoside (Ara-A)	Sigma-Aldrich	Cat#A5762
β-Nicotinamide adenine dinucleotide (NAD^+)	NEB	Cat#B9007S
Phenazine ethosulfate (PES)	Sigma-Aldrich	Cat#P4544
Thiazolyl blue tetrazolium bromide (MTT)	Sigma-Aldrich	Cat#M2128
SYBR Green	Sigma-Aldrich	Cat# S9430
Alcohol dehydrogenase (Adh)	Sigma-Aldrich	Cat#A3263
PARG enzyme	Trevigen	N/A
Exonuclease III	NEB	Cat#M0206S
APE1	NEB	Cat#M0282S
Uracil-DNA Glycosylase (UDG)	NEB	Cat#M0280S
Recombinant PARP1	This paper	N/A
Recombinant XRCC1	This paper & Caldecott et al., 1995	N/A
Recombinant DNA Polymerase Beta	This paper & Sam Wilson/ Caldecott et al., 1996	N/A
Recombinant DNA ligase IIIα	This paper & Caldecott et al., 1996	N/A
Critical commercial assays		
CellTiter-Glo	Promega	Cat#G7570
Experimental models: cell lines		
Human: hTERT RPE-1	ATCC	N/A
Human: hTERT RPE-1 *PARP1*$^{-/-}$	Hanzlikova et al., 2017	N/A
Human: hTERT RPE-1 *PARP2*$^{-/-}$	Hanzlikova et al., 2017	N/A
Human: hTERT RPE-1 *PARP1*$^{-/-}$ *PARP2*$^{-/-}$	Hanzlikova et al., 2017	N/A
Human: hTERT RPE-1 *XRCC1*$^{-/-}$	Hanzlikova et al., 2017	N/A
Human: hTERT RPE-1 *XRCC1*$^{-/-}$ *PARP1*$^{-/-}$	Hoch et al., 2017	N/A
Human: U2OS	ATCC	HTB-96
Human: U2OS *XRCC1*$^{-/-}$	(Polo et al., 2019)	N/A
Human: U2OS *PARP1*$^{-/-}$	Hanzlikova et al., 2017	N/A

(*Continued on next page*)

Continued

REAGENT or RESOURCE	SOURCE	IDENTIFIER
Human: U2OS *XRCC1*$^{-/-}$ + Myc-His-XRCC1	This paper	N/A
Human: U2OS *XRCC1*$^{-/-}$ + Myc-His-XRCC1-161-406	This paper	N/A
Human: TK6	ATCC	N/A
Human: TK6 *PARP1*$^{-/-}$	This paper	N/A
Human: TK6 *XRCC1*$^{-/-}$	This paper	N/A
Human: TK6 *XRCC1*$^{-/-}$ *PARP1*$^{-/-}$	This paper	N/A
Oligonucleotides		
For oligonucleotides see Table S1	This paper	N/A
Recombinant DNA		
pCD2E (Empty vector)	Caldecott et al., 1994	N/A
pCD2E-Myc-His-XRCC1	This paper	N/A
pCD2E-Myc-His-XRCC1-161-406	This paper	N/A
pET16b-XRCC1-His	Caldecott et al., 1995	N/A
Bacterial DNA Polymerase Beta expression	Sam Wilson/Caldecott et al., 1996	N/A
pET16b-His-DNA Ligase IIIα	Caldecott et al., 1996	N/A
GFP-PARP1	Shao et al., 2020	N/A
Software and algorithms		
Comet Assay IV software	Perceptive Instruments	N/A
Graph/statistical software	GraphPad Prism 9	https://www.graphpad.com/
Other		
Lipofectamine RNAiMAX Transfection Reagent	Thermofisher Scientific	Cat #13778100
Lipofectamine 2000 Transfection Reagent	Invitrogen	Cat #11668019
Neon Transfection System	Invitrogen	MPK5000
Amicon Ultra-0.5 Centrifugal Filter Unit	Millipore	Cat #UFC501024
Spin-X Centrifuge Tube Filter	Costar	Cat #8163
Fluoroskan Ascent FL	Thermofisher Scientific	N/A
His-Trap	GE Healthcare	GE29-0510-21
HiLoad 16/600 Superdex 200	GE Healthcare	GE28-9893-35
HiTrap Q	GE Healthcare	GE29-0513-25
HiTrap SP	GE Healthcare	GE29-0513-24
AKTA Pure FPLC system	GE Healthcare	N/A

RESOURCE AVAILABILITY

Lead contact

Further information and requests for resources and reagents should be directed to and will be fulfilled by the Lead Contact, K.W.Caldecott (k.w.caldecott@sussex.ac.uk).

Materials availability

All unique reagents (plasmids/cell lines etc) will be provided on request to academic laboratories without restrictions.

Data and code availability

All primary data are available on request to the lead contact. No code or large genomic/proteomic datasets are associated with this work.

EXPERIMENTAL MODEL AND SUBJECT DETAILS

Cell lines

Human hTERT RPE-1 cells (ATCC) and U2OS cells were incubated in a low oxygen (3%) incubator (37°C, 5% CO_2). RPE-1 cells were maintained in DMEM-F12 Glutamax 10% FBS supplemented with penicillin/streptomycin. U2OS were maintained in DMEM 10%

FBS supplemented with penicillin/streptomycin and L-glutamine. Human TK6 cells were cultured in 20% oxygen (37°C, 5% CO_2) in RPMI 1640 medium (Nacalai Tesque, Kyoto, Japan) supplemented with heat-inactivated horse serum (10%) (GIBCO, lot no. 2017-06), Sodium pyruvate (0.1 mM), L-glutamine, and penicillin/streptomycin. The *XRCC1*$^{-/-}$, *PARP1*$^{-/-}$ and *XRCC1*$^{-/-}$/ *PARP1*$^{-/-}$ RPE-1 cells, and U2OS *XRCC1*$^{-/-}$ cells, used in this study were generated and characterized previously (Hanzlikova et al., 2017; Hoch et al., 2017; Polo et al., 2019). U2OS *XRCC1*$^{-/-}$ cells complemented with full-length N-terminal Myc-His-tagged XRCC1 or truncated N-terminal Myc-His-tagged XRCC1$^{161\text{-}406}$ were generated as described below ("siRNA and cell transfection").

METHOD DETAILS

Chemicals

The PARP inhibitor KU 0058948 hydrochloride and PARG inhibitor were purchased from Axon (Cat#2001; CAS: 763111-49-5) and Tocris (PDD0017273; Cat#5952; CAS: 1945950-21-9), respectively. Both inhibitors were dissolved in dimethyl sulfoxide (DMSO) to a stock concentration of 10 mM and used at a final working concentration of 10 μM. Methyl methanesulfonate (MMS; Sigma-Aldrich, Cat#129925) was dissolved directly into culture medium and concentration used is as indicated in Figures. Recombinant PARG enzyme was from Trevigen.

Antibodies

Mouse anti-PARP1 monoclonal (Santa Cruz Cat#sc-8007; RRID:AB_628105) was employed at 1:500 rabbit polyclonal anti-PARP2 (Active Motif Cat#39743; RRID:AB_2793328) at 1:5000, rabbit anti-XRCC1 polyclonal (Novus; Cat#NBP1-87154; RRID:AB_11029388) at 1:5000, rabbit anti-DNA polymerase beta polyclonal (Millipore; Cat#6C0087) at 1:1000, rabbit anti-DNA ligase III polyclonal sera (TL25) at 1:5000, anti-poly-ADP-ribose binding reagent (Millipore; Cat# MABE1031; RRID:AB_2665467) at 1:10,000, mouse anti-alpha-Tubulin monoclonal (Sigma-Aldrich; Cat#T6074; RRID:AB_477582) at 1:10000, and rabbit anti-histone H3 polyclonal (Abcam; Cat#ab1791; RRID:AB_302613) at 1:10000.

siRNA and transfection

Non-targeting siRNA (ON-TARGETplus) and SMARTpool siRNA (25 nM) against PARP1 or PARP2 were reverse-transfected into cells using Lipofectamine® RNAiMAX (Invitrogen) according to the manufacturer's instructions. All experiments were carried out 72 hr post-transfection. U2OS *XRCC1*$^{-/-}$ cells complemented with full-length N-terminal Myc-His-tagged XRCC1 or truncated N-terminal Myc-His-tagged XRCC1$^{161\text{-}406}$ were generated by co-transfection with an empty vector (pCI-Puro) encoding resistance to puromycin and either pCD2E (empty vector), pcD2E-Myc-His-XRCC1, or pcD2E-Myc-His-XRCC1$^{161\text{-}406}$. Following transfection, cells were selected against puromycin (2 μg/ml) and after one week single colonies were isolated, amplified, and validated for expression of recombinant XRCC1.

Live cell imaging

The recruitment and exchange of GFP-tagged PARP1 was measured in wild-type and *Xrcc1*$^{-/-}$ MEFs as described previously (Shao et al., 2020) with minor modification. Briefly, ~1x10^4 MEFs were seeded on 35 mm diameter glass-bottom plate on day 1 and transiently transfected with GFP-PARP1 plasmid (1 μg) via Lipofectamine 2000 on day 2 (Invitrogen). All images were collected on day 4 (2 days after transfection) with a Nikon Ti Eclipse inverted microscope equipped with the Lu-N3 Laser Units and the A1 RMP confocal system (all from Nikon Inc, Tokyo, Japan). The micro-irradiation was generated by a 405 nm laser (energy level ~500 μW) in ~0.8 μm diameter nucleoli-free region of the nuclei. The GFP-PARP1 recruitment were measured in control untreated cells and cells treated with 0.3 mg/ml MMS (Sigma, Cat. 64294) for ≥ 60 min. Images were taken immediately following micro-irradiation (defined as 0 s) up to 1 minute with 2 s interval. The relative intensity of GFP-PARP1 foci is defined as the mean green fluorescent intensity at the micro-radiated area (0.8 μm diameter)/mean green fluorescent intensity in the entire nucleus. For FRAP, the cells were either untreated or pre-treated as indicated with 0.3 mg/ml MMS for 0-30 minutes or ≥ 60 minutes before micro-irradiation. A confined nucleoli-free area of ~0.8 μm diameter in the nucleus was bleached with a 488 nm laser targeting GFP (energy level = 217 μW) and images were taken every other second to 1 minute. The recovery curve was plotted as the percentage of GFP intensity (damage region)/GFP intensity (whole nucleus) at the given time points versus before bleaching of each given cell. At least 6 cells were collected for recruitment analysis of each condition and at least 10 cells were collected for FRAP assay of each condition.

XRCC1 protein complexes and PARP1 activity, *in vitro*.

Recombinant histidine-tagged human XRCC1 (XRCC1-His) and DNA ligase IIIα (His-LIG3), and untagged DNA polymerase β (POLβ), were expressed in and purified from 1-2 l of *E.coli* culture essentially as described previously (Caldecott et al., 1995, 1996), using an AKTA Pure FPLC system. In brief, recombinant XRCC1-His and His-LIG3 were purified by sequential metal-chelate affinity chromatography and gel filtration using 1ml HisTrap (GE Healthcare) HiLoad 16/600 Superdex 200 (GE Healthcare), respectively. Recombinant human POLβ was purified by sequential anion exchange, cation exchange, and gel filtration using HiTrap Q HP (2x1ml; GE healthcare), HiTrap SP (1ml; GE Healthcare) and HiLoad 16/600 Superdex 200 columns.

PARP1 reactions (20 μL) were incubated at room temp for 5 min and contained 50 mM Tris-HCl pH7.9, 100 mM NaCl, 10 mM $MgCl_2$, 100 μg/ml BSA, 0.3 μM each of the indicated recombinant proteins, 10 μM NAD^+ (where indicated), and 0.15 μM of fluorescein labeled

double-hairpin duplex substrate containing a single uracil residue (IDT; Integrated DNA Technologies). Reactions were terminated by the addition of SDS-PAGE sample buffer and heating at 95°C. for 5 minutes. The duplex hairpin substrate was generated by heating/cooling the single strand oligonucleotide 5′- GCACGGCGCATCAGCTGCAGAACAACTGCAGCTGATGCGC/deoxyU/GTGCGGATCCGGTGCAAC/iFluorT/AAGCACCGGATCC-3′ to form the duplex followed by ligation of the residual nick with T4 ligase and gel purification. The substrate possessed a single internal fluorescein dT residue for visualization/purification. The substrate was pre-incubated or not as indicated with uracil DNA glycosylase and AP endonuclease (UDG/APE1; 1.2 U each; NEB) to create a SSB intermediate of BER (cleaved abasic site) prior to the addition, where indicated, of recombinant PARP1, XRCC1, POLB, LIG3.

Gene-targeting XRCC1 in TK6 cells

XRCC1 was disrupted with knockout constructs prepared using primers 5′-GTAGTAAAAGACAGATGCCCACAGTCCACA-3′ and 5′-CTGGCTGCTGCAGGACACGACATGGCGGAG-3′ for the left arm and 5′-ACTCACTGTGCAGAAAATCTTCTCAAGGCA-3′ and 5′-AACCACCATACCTGGCTATTATTCTTTAAA-3′ for the right arm. The TALEN vector was designed to recognize the following sequences: 5′-TGACATGCCGGAGATCCG-3′ and 5′-GTCCTGCAGCAGCCAGGA-3′. *PARP1* was disrupted with knockout constructs prepared using primers 5′-TGGGGAGTAGTGCTTTGTTTGGATATATCC-3′ and 5′-CTGGAGAATCAAACAGACAGCAATGCTCAT-3′ for the left arm and 5′-GTAAGATCTTGGGGGCCCAGATCCCTGAAC-3′ and 5′-CTTAAATTCCAAATGGCTGGCAACCTACCT-3′ for the right arm. We used CRISPR/Cas9 and the guide sequence 5′-GAAGTACGTGCAAGGGGTGTATGG-3′ to facilitate gene targeting. For gene targeting, we used the maker genes *DT-ApA/NEO*^{R} (provided by the Laboratory for Animal Resources and Genetic Engineering, Center for Developmental Biology, RIKEN Kobe; http://www.clst.riken.jp/arg/cassette.html) and *DT-ApA/PURO*^{R} digested with *Apa*I and *Afl*II. Wild-type TK6 cells were transfected with the above-mentioned targeting vectors (2 μg), and the expression vector (10 μg) for TALEN or CRISPR (pX330; Addgene, US), employing the NEON transfection system (Invitrogen, CA) at 1500 V 20 msec. *β-actin* transcripts were used as a positive control for the RT-PCR analysis using primers 5′-GATGGTGGGCATGGGTCAGAAGGATTCC-3′ and 5′-GTCCAGGGCGAGGTAGCACAGCTTCTC-3′.

Alkaline comet assays

Cells were treated with the indicated concentration of MMS in the presence/absence of 10 μM PARP inhibitor (KU0058948) for 15 min at 37°C in medium, and alkaline comet assays conducted essentially as described previously (Breslin et al., 2006). In brief, following the indicated treatment of 5x10^4 cells in suspension (1 ml) with MMS , cells were washed once in ice-cold PBS and resuspended in 0.45 mL PBS. Aliquots (0.15 ml) of the cell suspension mixed with an equal volume of low gelling agarose (at 42°C), spread on agarose pre-coated frosted slides and set on ice, and then lysed in alkaline lysis buffer (2.5M NaCl, 100 mM EDTA, 10 mM Tris-Cl, 1% v/v DMSO, 1% v/v Triton X-100, pH 10) at 4°C for 60 min. Samples were then pre-incubated for 45 min and subject to electrophoresis at 12V for 25 min in electrophoresis buffer (50mM NaOH, 1mM EDTA, 1% DMSO, pH13) at 4°C. Slides were neutralized in 0.4M Tris-Cl pH 7.4 prior to staining in PBS containing SYBR Green (Sigma, 1:10000 dilution) and 0.04 mg/ml p-Phenyledenediamine dihydrochloride anti-fade (Fisher 417481000). Comet tail moments (an arbitrary-unit measure of DNA strand breaks) from 50-100 cells per sample were scored blinded and using automated Comet Assay IV software (Perceptive Instruments, UK).

Cell survival assays

For RPE-1 and U2OS cells we conducted clonogenic survival assays. Cells were plated in 10 mm plates and 4 h later treated with indicated concentrations of MMS for 30 min or with CPT continuously. Cells were rinsed twice with PBS, incubated in drug-free medium for 10 days, and then fixed in 100% ethanol and stained with 0.05% crystal violet. The surviving fraction at each dose was calculated by dividing the average number of colonies (defined as > 50 cells) in treated dishes by the average number in untreated dishes. For TK6 cells, we employed a liquid-culture cell survival assay, as described previously (Tsuda et al., 2017). 1x10^6 cells were suspended in PBS (1 ml) containing horse serum (1%) and treated with MMS for 1 h. Treated cells (10 μl) were transferred to culture medium (1 ml) and cultured for 72 h. The incubated cells (100 μl) were transferred to 96-well plates, and the amount of ATP was measured using CellTiter-Glo (Promega; Cat# G7570), according to the manufacturer's instructions. Luminescence was measured using the Fluoroskan Ascent FL (Thermo Fisher Scientific Inc., Waltham, MA).

Chromatin retention assay & immunoblotting

RPE-1, U2OS or human fibroblast cells were harvested and lysed in lysis buffer containing 100 mM, 150 mM or 150 mM KCl, respectively, and 50 mM HEPES pH 7.4, 2.5 mM $MgCl_2$, 5 mM EDTA pH 8, 3 mM dithiothreitol (DTT), 0.5% Triton X-100, 10% glycerol, and protease inhibitor cocktail (Roche) for 45 min on ice. Soluble and chromatin-bound proteins were separated by centrifugation (15 min, 16,000 g). The pellet containing the detergent insoluble material (including chromatin) was washed twice in lysis buffer and then subjected to sonication to shear the DNA. The soluble and chromatin extracts were mixed with SDS-PAGE sample buffer and heated for 10 min at 95°C. For protein levels in whole cell extracts (WCE), cells were lysed directly in SDS-PAGE sample buffer and heated as above. All protein extracts were subjected to SDS-PAGE followed by protein transfer onto nitrocellulose membrane and western blotting with the indicated antibodies.

CellPress
OPEN ACCESS

NAD^+ measurements.

NAD^+ levels in RPE-1 cells were determined by a chromogenic assay as described before (Baker et al., 2016). Briefly, cells were treated with 0.3 mg/ml MMS in culture media at 37°C for the indicated time, washed with PBS and scraped in PBS supplemented with 100 μM PARG inhibitor (PDD00017273, Sigma), 40 μM PARP inhibitor (KU 0058948, Axon), and cOmplete protease inhibitors (04693132001, Roche). Cell pellets were resuspended in lysis buffer (20 mM sodium bicarbonate, 100 mM sodium carbonate, 0.5% Triton X-100, 10 mM nicotinamide, 100 μM PARG inhibitor, 40 μM PARP inhibitor, and cOmplete protease inhibitors, pH 10.3) and lysed by two freeze/thaw cycles. Protein concentration was normalized, lysates were transferred to a 10,000 MWCO centrifugal filter (UFC501024, Merck) and centrifuged at 14,000 × g at 4 °C for 30 min. Half of each lysate was incubated at 60 °C for 30 min to decompose NAD^+. Samples were incubated in cycling buffer [100 mM tricine-NaOH (pH 8), 4 mM EDTA, 40 mM NaCl, 1.66 mM phenazine ethosulfate (PES), 0.42 mM thiazolyl blue tetrazolium bromide (MTT), 10% ethanol] at 37 °C for 5 min, and 10 U/ml alcohol dehydrogenase, reconstituted in 100 mM tricine-NaOH (pH 8), was added to drive a cycling reaction at 37 °C for 40 min. The reaction was terminated by addition of NaCl (2 M final concentration) and samples were centrifuged at 14,000 × g at 4 °C for 5 min. Reduced MTT was resuspended in 100% ethanol and the absorbance was measured at 560 nm. NAD^+ concentrations were calculated by subtracting the absorbance of samples with thermal decomposition prior to the cycling reaction from those samples without thermal decomposition prior to the cycling reaction.

QUANTIFICATION AND STATISTICAL ANALYSIS

Data were examined for statistical significance using GraphPad Prism 9 using one- or two-way ANOVA with appropriate post hoc tests, as indicated in the figure legends. For survival assays, plotted data are mean (+/−SEM) surviving fraction (%) calculated from the indicated number (N) of independent biological replicate experiments. For comet assays, plotted data are the comet tail moments of every individual cell in each of the indicated number (N) of independent experiments. Note that for the comet assays, individual cells within each experiment are technical replicates, and independent experiments are biological replicates.

Molecular Cell, Volume *81*

Supplemental information

XRCC1 prevents toxic PARP1 trapping during DNA base excision repair

Annie A. Demin, Kouji Hirota, Masataka Tsuda, Marek Adamowicz, Richard Hailstone, Jan Brazina, William Gittens, Ilona Kalasova, Zhengping Shao, Shan Zha, Hiroyuki Sasanuma, Hana Hanzlikova, Shunichi Takeda, and Keith W. Caldecott

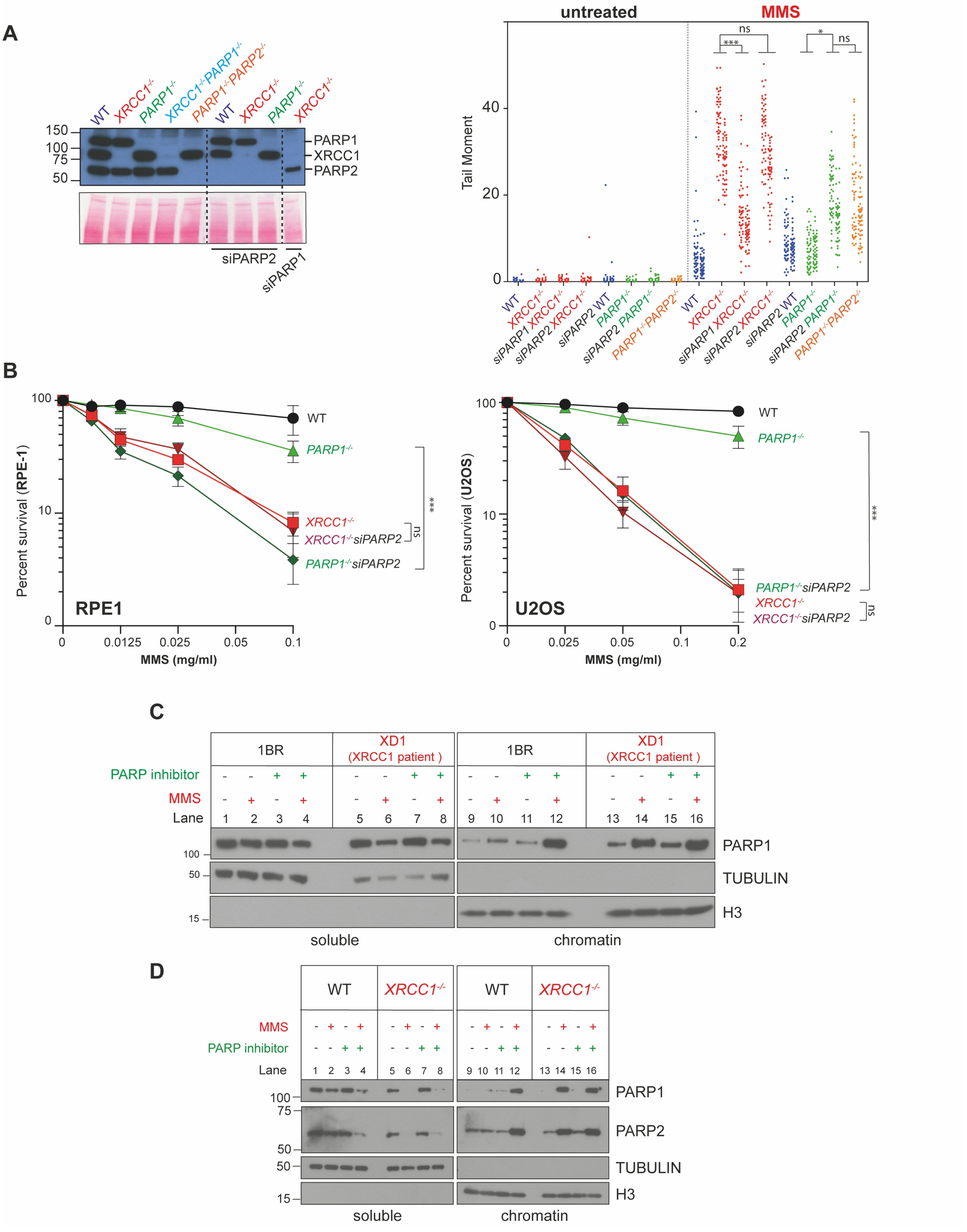

Figure S1. PARP1 but not PARP2 is responsible for the accumulation of SSBs in *XRCC1-/-* RPE-1 cells (see also Figure1 & Figure 2). A, *Left,* PARP1, PARP2, and XRCC1 levels in the indicated gene edited and/or siRNA treated cell lines. *Right*, Strand breaks quantified by alkaline comet assays in untreated or MMS-treated (0.1 mg/ml MMS, 15 min) wild-type (WT) and the indicated RPE1-cell lines, with or without prior treatment with PARP1/PARP2 siRNA. Data plotted are the individual comet tail moments of 50 cells per sample per experiment, for two independent experiments, with tail moments for each experiment plotted vertically and each experiment plotted side by side. Statistical significance was ascertained by one-way ANOVA, with Sidak's multiple

comparisons test (*p≤0.05; ***p≤0.001). **B**, Clonogenic survival of wild-type (WT) and the indicated gene-edited RPE-1 (*left panel*) or U2OS (*right panel*) cells after mock-treatment or treatment with PARP2 siRNA and with the indicated concentrations of MMS for 30 min, followed by incubation in drug-free medium for 10-14 days. Data are the mean (+/-SEM) of three independent experiments. Statistical significance was assessed by two-way ANOVA with Tukey's multiple comparisons test (ns, not significant; *p≤0.05; ***p≤0.001). **C,** PARP1 levels in cell-equivalent aliquots of soluble and chromatin-containing fractions of wild-type (1BR) and *XRCC1*-mutated patient (XD1) primary human fibroblasts, measured by western blotting **D**, PARP1 and PARP2 levels in cell-equivalent aliquots of soluble and chromatin-containing fractions of wild-type (WT) and *XRCC1*$^{-/-}$ RPE-1 cells, measured by western blotting. Cells were incubated or not with 10 μM PARP inhibitor (KU0058948) and/or MMS (0.1 mg/ml) for 1h as indicated, prior to subcellular fractionation.

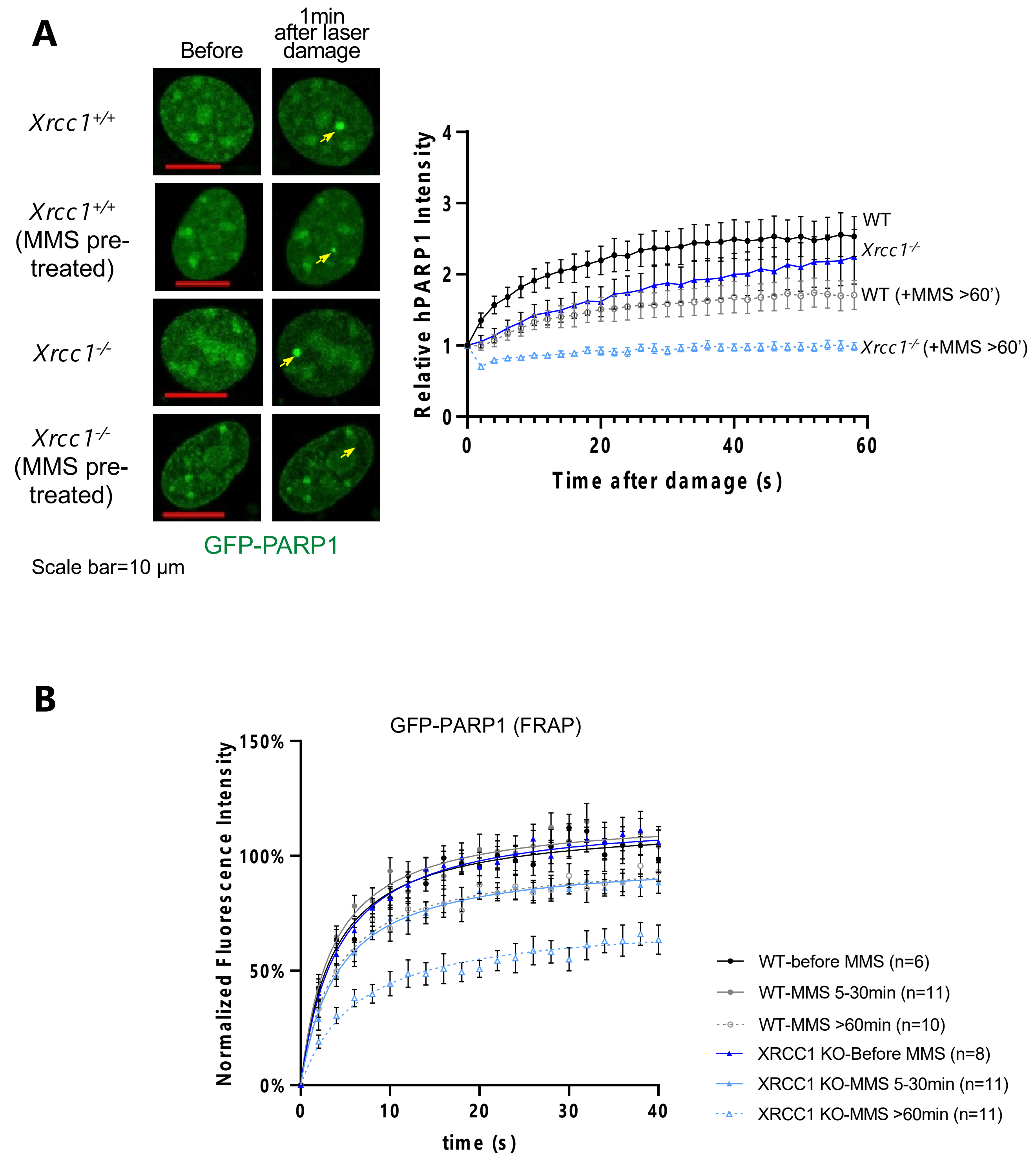

Figure S2. Reduced GFP-PARP1 mobility in MMS-treated *Xrcc1*$^{-/-}$ MEFs. A, Wild type (*Xrcc1*$^{+/+}$) and *Xrcc1*$^{-/-}$ MEFs expressing GFP-tagged PARP1 were pre-treated or not with 0.3 mg/ml MMS ≥60 min and then irradiated with 405 nm laser (yellow arrow). GFP-PARP1 recruitment at the site of damage was monitored for 60 sec following irradiations. **B,** Wild type and *Xrcc1*$^{-/-}$ MEFs expressing GFP-tagged PARP1 were pre-treated or not with 0.3 mg/ml MMS for 60 min, photobleaching by 488nm laser and monitoring of fluorescence recovery was conducted before, 5-30min after adding 0.3 mg/ml MMS, or ≥60min after adding MMS. Exchanging curves were fitted using one site ligand specific binding model using the Prism software suite. Data are representative of two independent experiments.

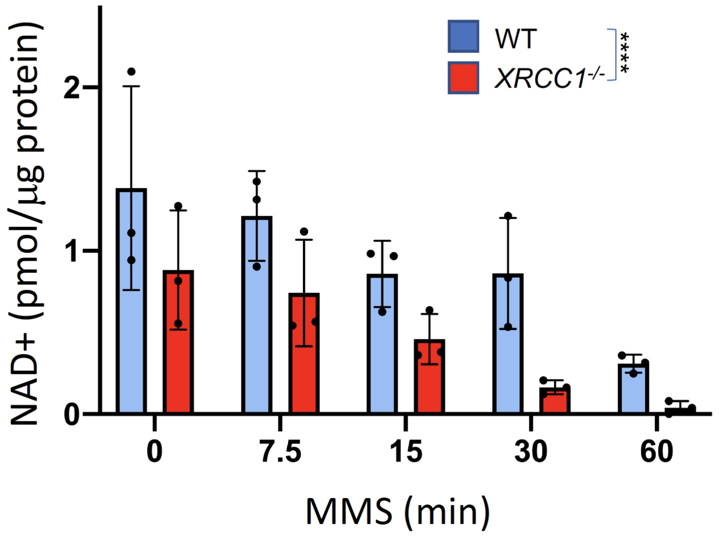

Figure S3. Increased PARP1-dependent NAD$^+$ depletion in *XRCC1$^{-/-}$* RPE1 cells, during BER (see also, Figure 4). The indicated wild type and gene-edited RPE1 cells were treated with 0.3mg/ml MMS for the indicated periods and NAD$^+$ levels present in protein extracts quantified chromogenically. Data are the mean of three independent experiments and statistical significance was assessed by 2-way ANOVA.

Supplementary Table S1; oligonucleotides employed in this study

ON-TARGETplus Human PARP1 siRNA (SMARTPool)	Dharmacon	L-006656-03-0005
ON-TARGETplus Human PARP2 siRNA (SMARTPool)	Dharmacon	L-010127-02-0020
ON-TARGETplus Non-targeting Pool	Dharmacon	D-001810-10
Double hairpin substrate 5'-GCACGGCGCATCAGCTGCAGAACAACTGCAGCTGATGCGC/deoxyU/GTGCGGATCCGGTGCAAC/iFluorT/AAGCACCGGATCC-3'	Integrated DNA Technologies (IDT)	N/A
TK6 XRCC1 knockout construct, left arm: 5'-GTAGTAAAAGACAGATGCCCACAGTCCACA-3' and 5'-CTGGCTGCTGCAGGACACGACATGGCGGAG-3'	This paper	N/A
TK6 XRCC1 knockout construct, right arm 5'-ACTCACTGTGCAGAAAATCTTCTCAAGGCA-3' and 5'-AACCACCATACCTGGCTATTATTCTTTAAA-3'	This paper	N/A
TK6 PARP1 knockout construct, left arm 5'-TGGGGAGTAGTGCTTTGTTTGGATATATCC-3' and 5'-CTGGAGAATCAAACAGACAGCAATGCTCAT-3'	This paper	N/A
TK6 PARP1 knockout construct right arm: 5'-GTAAGATCTTGGGGGCCCAGATCCCTGAAC-3' and 5'-CTTAAATTCCAAATGGCTGGCAACCTACCT-3'	This paper	N/A
β-actin (forward) 5'-GATGGTGGGCATGGGTCAGAAGGATTCC-3'	This paper	N/A
β-actin (reverse) 5'-GTCCAGGGCGAGGTAGCACAGCTTCTC-3'	This paper	N/A
